# Progression of the pluripotent epiblast depends upon the NMD factor UPF2

**DOI:** 10.1242/dev.200764

**Published:** 2022-11-07

**Authors:** Jennifer N. Chousal, Abhishek Sohni, Kristoffer Vitting-Seerup, Kyucheol Cho, Matthew Kim, Kun Tan, Bo Porse, Miles F. Wilkinson, Heidi Cook-Andersen

**Affiliations:** ^1^ Department of Obstetrics, Gynecology and Reproductive Sciences, School of Medicine, University of California, San Diego, La Jolla, CA 92093, USA; ^2^ Department of Molecular Biology, University of California, San Diego, La Jolla, CA 92093, USA; ^3^ The Bioinformatics Centre, Department of Biology and Biotech Research & Innovation Centre, University of Copenhagen, 2200 Copenhagen, Denmark; ^4^ Section for Bioinformatics, Health Technology, Technical University of Denmark (DTU), 2800 Kongens Lyngby, Denmark; ^5^ The Finsen Laboratory, Rigshospitalet, Faculty of Health Sciences, University of Copenhagen, DK2200 Copenhagen, Denmark; ^6^ Biotech Research and Innovation Center (BRIC), University of Copenhagen, 2200 Copenhagen, Denmark; ^7^ Novo Nordisk Foundation Center for Stem Cell Biology, DanStem, Faculty of Health Sciences, University of Copenhagen, 2200 Copenhagen, Denmark; ^8^ Institute of Genomic Medicine, University of California, San Diego, La Jolla, CA 92093, USA

**Keywords:** Blastocyst, Epiblast, RNA decay, Nonsense-mediated RNA decay

## Abstract

Nonsense-mediated RNA decay (NMD) is a highly conserved RNA turnover pathway that degrades RNAs harboring in-frame stop codons in specific contexts. Loss of NMD factors leads to embryonic lethality in organisms spanning the phylogenetic scale, but the mechanism remains unknown. Here, we report that the core NMD factor, UPF2, is required for expansion of epiblast cells within the inner cell mass of mice *in vivo*. We identify NMD target mRNAs in mouse blastocysts – both canonical and alternatively processed mRNAs – including those encoding cell cycle arrest and apoptosis factors, raising the possibility that NMD is essential for embryonic cell proliferation and survival. In support, the inner cell mass of *Upf2*-null blastocysts rapidly regresses with outgrowth and is incompetent for embryonic stem cell derivation *in vitro*. In addition, we uncovered concordant temporal- and lineage-specific regulation of NMD factors and mRNA targets, indicative of a shift in NMD magnitude during peri-implantation development. Together, our results reveal developmental and molecular functions of the NMD pathway in the early embryo.

## Teaser

**Highlighted Article:** The RNA decay factor, UPF2, drives developmental progression of the mouse inner cell mass, with its loss leading to a deficit of epiblast cells and peri-implantation embryo lethality.

## INTRODUCTION

During preimplantation development, the embryo undergoes two differentiation steps, culminating in the formation of an implantation-competent blastocyst. The embryo first divides into the inner cell mass (ICM) and trophectoderm (TE) at approximately embryonic day (E) 3.25, and the ICM further differentiates to form the epiblast and primitive endoderm (PrE) (Rossant, 2018). Immediately after, during peri-implantation development (E4.0-E5.5), the blastocyst hatches from the zona pellucida and attaches to the uterine wall to initiate implantation. The TE invades the endometrium to establish the placenta, the PrE forms an epithelium that encircles the embryo and will form the yolk sac, and the pluripotent epiblast rapidly proliferates in preparation for gastrulation (Rossant, 2018; Morris et al., 2012a). During post-implantation development (>E5.5), the embryonic structure is solidified, setting the stage for the remainder of the pregnancy. Although both preimplantation and post-implantation development have been extensively studied, peri-implantation development remains less well understood, largely because of challenges in obtaining embryos at this stage and the limited cell numbers available for analysis (Bedzhov and Zernicka-Goetz, 2014). In this study, we identify a molecular pathway crucial for normal mammalian peri-implantation development.

The majority of studies examining factors important for early embryo development have focused on transcription (Chazaud and Yamanaka, 2016; Rossant, 2018). Given that steady-state mRNA levels are dictated by not only transcription rate, but also mRNA turnover rate, it is important to consider the possibility that embryonic development is also controlled by regulated RNA turnover. In support of this possibility, it has been shown that regulated RNA turnover can influence – and even drive – precise temporal and spatial control of gene expression, as well as specific biological functions (Alonso, 2012; Schoenberg and Maquat, 2012; Svoboda et al., 2015; Tan and Wilkinson, 2022; Yamada and Akimitsu, 2019). RNA turnover confers essential properties anticipated to be selected for over evolutionary time (Alonso, 2012). One such property is efficient gene silencing. Transcriptional downregulation is not sufficient to achieve this goal; it is also necessary to destabilize the pre-existing mRNA pool. Another property conferred by regulated RNA decay is dramatic shifts in gene expression. For example, simultaneous upregulation of transcription rate and decreased RNA destabilization rate can induce very high levels of mRNA (Tan and Wilkinson, 2022).

One of the most well-studied RNA turnover mechanisms is the highly conserved nonsense-mediated RNA Decay (NMD) pathway. NMD was originally discovered by virtue of its ability to degrade aberrant RNAs harboring premature termination codons (PTCs), thereby protecting cells from truncated dominant-negative proteins (Chang et al., 2007). Subsequently, it was discovered that NMD degrades subsets of normal RNAs, with loss or disruption of NMD leading to dysregulation of 5-20% of the normal transcriptome in species spanning the phylogenetic scale (Chan et al., 2007; Gatfield et al., 2003; He et al., 2003; Lelivelt and Culbertson, 1999; Mendell et al., 2004). This raised the possibility that NMD regulates numerous biological processes, which has been supported by many subsequent studies (Han et al., 2018; Jaffrey and Wilkinson, 2018; Kurosaki et al., 2019; Lejeune, 2017; Nasif et al., 2018). However, the full reach of the regulatory capacity of NMD has yet to be uncovered.

A large body of research has demonstrated that loss or perturbation of NMD factors cause early embryo lethality (Alonso and Akam, 2003; Anastasaki et al., 2011; Casadio et al., 2014; Longman et al., 2007; Mao et al., 2015; Metzstein and Krasnow, 2006; Silver et al., 2010). In mice, null mutations in any of several different NMD factor genes – *Upf1*, *Upf2*, *Upf3a*, *Smg1* and *Smg6* – cause lethality during peri-implantation (and possibly the early stages of post-implantation) development (Hwang and Maquat, 2011; Li et al., 2015; Mao et al., 2015; McIlwain et al., 2010; Medghalchi et al., 2001; Shum et al., 2016; Weischenfeldt et al., 2008). However, the nature of the defects preceding lethality and the molecular mechanisms responsible are poorly understood. Here, we investigate the role of NMD in early embryo development using a mouse model lacking the core NMD factor UPF2.

## RESULTS

### Upf2 is required for mouse peri-implantation embryo viability

**Fig. 1.**
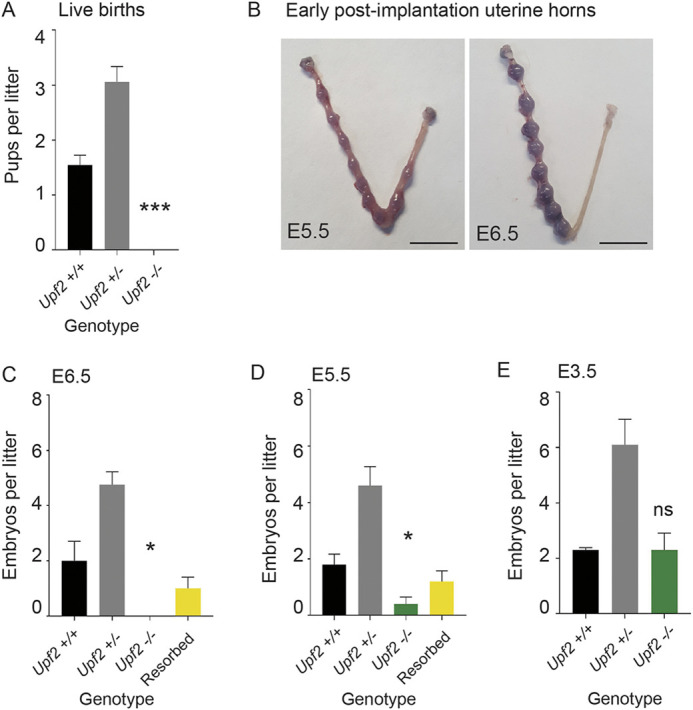
**Loss of *Upf2* results in peri-implantation embryonic lethality.** (A) Live births per litter, from *Upf2*^+/−^ crosses (***P<0.0001, two-tailed Student's *t*-test *Upf2^+/+^* versus *Upf2^−/−^*). *Upf2*^+/−^:*Upf2*^+/+^ pups were present at a 2:1 Mendelian ratio (*n*=33 mating pairs). (B) Uterine horns of *Upf2*^+/−^ females (mated with *Upf2*^+/−^ males) at E5.5 (left) and E6.5 (right). Implantation sites are visualized by blue dye. (C,D) Early post-implantation embryos were isolated from the horns of *Upf2*^+/−^ females (mated with *Upf2*^+/−^ males) at (C) E6.5 (*n*=26 embryos, *n*=4 mice) and (D) E5.5 (*n*=34 embryos, *n*=5 mice), presented as embryos per litter. No *Upf2*-null embryos were present at E6.5 (*P=0.03, two-tailed Student's *t*-test *Upf2*^+/+^ versus *Upf2*^−/−^), and two *Upf2*-null embryos were present at E5.5 (*P=0.01, two-tailed Student's *t*-test *Upf2*^+/+^ versus *Upf2*^−/−^). (E) Blastocysts were flushed from the uterine horns of superovulated *Upf2*^+/−^ females (mated with *Upf2*^+/−^ males) at E3.5 (*n*=63 embryos, *n*=7 mice; *P*=0.50, two-tailed Student's *t*-test *Upf2^+/+^* versus *Upf2^−/−^*). *Upf2-*null blastocysts were present at the expected Mendelian ratio. Data are mean±s.e.m. Scale bars: 1 cm.

To knockout *Upf2* in the early embryo, we generated global heterozygous (*Upf2^+/−^*) mice by crossing *Upf2*-floxed (*Upf2^Fl/Fl^*) mice harboring loxP sites in introns 1 and 3 (Weischenfeldt et al., 2008) with mice expressing Cre recombinase driven by the *E2a* promoter, which is expressed early in preimplantation development (Fig. S1A). Recombination results in deletion of exons 2 and 3 (Fig. S1B), which generates a truncated protein lacking NMD activity (Weischenfeldt et al., 2008). *Upf2-*heterozygous (*Upf2^+/−^*) crossings produced no *Upf2-*null (*Upf2^−/−^*) pups, consistent with embryonic lethality (Weischenfeldt et al., 2008). *Upf2-*heterozygous and wild-type (*Upf2^+/+^*) pups were born at a 2:1 ratio (Fig. 1A), consistent with Mendelian inheritance. The mean litter size was 4.6 (±2; ±s.e.m.) pups compared with 7.5 (±2) for wild-type mice of the same strain.

It was previously reported that loss of *Upf2* leads to embryonic lethality in mice by E9.5 or earlier, with fewer *Upf2-*null embryos isolated at E3.5 than expected (Weischenfeldt et al., 2008). To precisely define the timing of embryo lethality, we isolated early post-implantation embryos at various time points from *Upf2*-heterozygous crosses (Fig. 1B). At E5.5, the earliest time that implantation can be accurately assessed, only two *Upf2*-null embryos were recovered (*n*=34 embryos, five matings) (Fig. 1B,D). At E6.5, no Upf2-null embryos were recovered (*n*=26 embryos, five matings) (Fig. 1B,C). Implantation sites and resorption sites per female from *Upf2*-heterozygous crosses were within the normal range observed for wild-type C57/BL6 mice (Flores et al., 2014) and not different between E5.5 and E6.5 (Fig. 1B-D; Fig. S1C). Although it was not possible to genotype resorbed embryos because of the limited amount of embryonic DNA recovered, these findings suggest that *Upf2*-null embryos do not have an abnormally high resorption rate.

To investigate whether loss of *Upf2* also influences preimplantation development, we examined E3.5 blastocyst-stage embryos from heterozygous matings. The frequency of *Upf2*-null blastocysts was consistent with a normal Mendelian ratio (Fig. 1E), demonstrating no obvious defect in development to the blastocyst stage. The small proportion of immature *Upf2-*null embryos relative to expanded blastocysts did not significantly deviate from littermate controls (*P*=0.48, χ^2^ test) (Fig. S1D). Similarly, *in vitro* development of *Upf2-*null embryos from zygote to blastocyst occurred at a rate indistinguishable from controls (*P*=0.16) (Fig. S1E,F). Together, these data demonstrate that UPF2 is not required for development to the blastocyst stage but plays an essential role in the steps that immediately follow, during peri-implantation development.

### Transcriptome analysis of *Upf2*-null mouse blastocysts

**Fig. 2.**
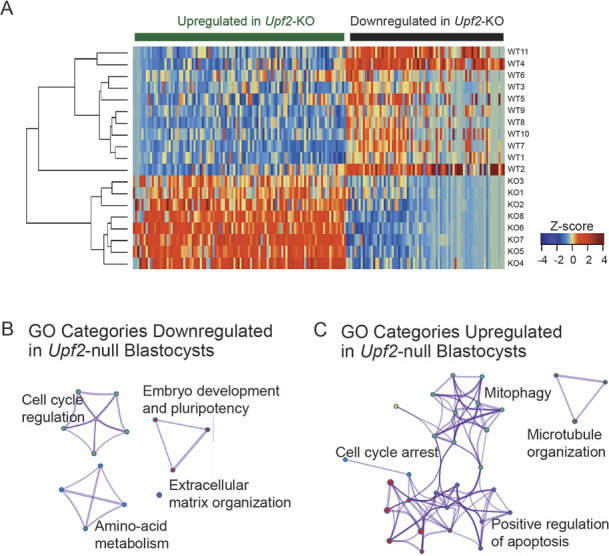
**Loss of *Upf2* leads to dysregulation of mRNAs associated with embryo development and cell survival.** (A) Heatmap of differentially expressed genes in *Upf2-*null blastocysts compared with controls. (B) Gene ontology (GO) categories enriched in genes downregulated in *Upf2-*null blastocysts (*n*=76) compared with controls, including regulation of development, pluripotency and metabolism. (C) GO categories enriched in upregulated genes (*n*=102) demonstrate that UPF2 regulates genes important for cell survival/apoptosis, cell cycle progression, mitophagy and microtubule organization.

To investigate the molecular mechanism by which NMD acts in the early embryo, we performed RNA-sequencing (RNA-seq) analysis on *Upf2*-null and littermate control blastocysts at E3.25 (Fig. S2A). We chose this time point to capture any molecular defects that might be responsible for the overt embryo phenotypic defects that likely arise in the late blastocyst. Consistent with no overt phenotypic defects in the early blastocyst, plotting the principal components that represent the most variance in our dataset did not lead to clustering of *Upf2^−/−^* (*n*=8) and *Upf2^+/+^* (*n*=11) samples. Instead, we found that loss of UPF2 affected the expression of a specific cohort of mRNAs (Fig. S2B). In total, 178 genes were significantly differentially expressed between *Upf2-*null and control blastocysts [adjusted *P*-value (*Q*)<0.1; Fig. 2A; Table S2].

The genes significantly downregulated in *Upf2-*null blastocysts (76) are enriched for functions important for growth and development (Fig. 2B), with known roles in regulating ‘embryo development and pluripotency’ (e.g. the *Nanog*, *Fgf4*, *Rspo1*, *Gata1* and *Phlda2* genes), raising the possibility that UPF2 plays a role within the pluripotent epiblast. Also highly represented are genes involved in ‘extracellular matrix organization’ (*Col5a1*, *Ltbp3*, *Matn3* and *Crtap*), which are intimately linked to peri-implantation development (Smyth et al., 1999; Yang et al., 2002). Other regulated genes are involved in positive regulation of the cell cycle (*Bora*, *Fgf4*, *Kif23*, *Nanog*, *Spast* and *Rspo1*) and amino-acid metabolism (*Gfpt1*, *Odc1*, *Crtap* and *Qars*), the latter of which is known to be important for energy production in the embryo (Gardner et al., 2001). Together, these results raised the possibility that NMD has roles in early embryo developmental progression and cell proliferation.

The genes upregulated in *Upf2-*null blastocysts (102) are enriched for ‘cell cycle arrest’ (*Gadd45a*, *Gorab*, *Mdm4*, *Rassf1* and *Txnl4b*) functions (Fig. 2C), which provides further support for the notion that NMD drives the proliferation of early embryonic cells. Other upregulated genes are also linked to cell proliferation, including those involved in ‘microtubule organization’ (*Sdhaf2*, *Smim20*, *Fis1* and *Coa4*). Also enriched are genes involved in ‘apoptosis’ (*Atf4*, *Ddit3*, *Fis1*, *Gadd45a*, *Gorab*, *Ip6k2*, *Mdm4*, *Rassf1* and *Tex261*) and apoptosis-related events, including ‘mitophagy’ (*Fis1*, *Mtcl1* and *Zdhhc4*) (Fig. 2C), providing further support that UPF2 promotes cell survival. In total, three of the five most highly enriched Gene Ontology (GO) categories involve either cell proliferation or apoptosis.

### Identification of NMD target mRNAs in the mouse blastocyst

The NMD pathway is thought to regulate biological and developmental systems by degrading specific mRNAs (Han et al., 2018; Lou et al., 2014; Lykke-Andersen and Jensen, 2015). Thus, it is crucial to define NMD target mRNAs. Although many high-confidence NMD target mRNAs have been identified in immortalized cell lines (Huang et al., 2011; Karam et al., 2015; Lou et al., 2014; Lykke-Andersen et al., 2014; Schmidt et al., 2015; Tani et al., 2012; Wang et al., 2011; Yepiskoposyan et al., 2011), few have been identified in normal cells. A further issue is that the repertoire of NMD target mRNAs may differ between biological contexts (Huang et al., 2011), necessitating identification of NMD targets in the specific cell type of interest*.*

To identify high-confidence NMD target mRNAs in blastocysts, we integrated three complementary approaches. First, we defined NMD-destabilized mRNAs by inferring RNA stability rate from steady-state RNA data. In this approach, intronic and exonic reads serve as a proxy for pre- and spliced mRNAs, respectively, allowing for the inference of transcription and RNA decay rates (Alkallas et al., 2017). Comparing these values in *Upf2-*null versus control embryos, we calculated a differential stability score (DSS) that reflected the difference in RNA stability as a result of *Upf2* loss. We identified 673 genes encoding mRNAs stabilized in *Upf2-*null embryos (DSS>0.1) (Fig. S2C; Table S2). Sixteen of these were also significantly upregulated at the steady-state level. mRNAs both upregulated and stabilized were enriched for elevated stability scores (Fig. S2C; Table S2). Although the proportion of stabilized mRNAs also upregulated at steady-state is modest, this is in alignment with a previous study showing that many mRNAs stabilized in NMD-deficient cells are not upregulated at steady state, likely due to transcriptional feedback mechanisms (Tani et al., 2012).

As a second approach to identify bona fide NMD target mRNAs, we screened mRNAs upregulated upon *Upf2* loss for features known to elicit NMD. The NMD-inducing feature that most consistently triggers NMD is an exon-exon junction downstream of the stop codon (dEJ) defining the main open reading frame (ORF) (Lykke-Andersen and Jensen, 2015). RNAs both upregulated and stabilized in *Upf2*-null blastocysts were enriched for dEJs (40% of upregulated and stabilized RNAs versus 17% of all RNAs) (Fig. S2D; Table S2), suggesting that the dEJ elicits the decay of many NMD targets in the blastocyst. mRNAs classified as either stabilized or upregulated, but not both, had intermediate dEJ enrichment (Fig. S2D; Table S2). Another feature that elicits NMD in some contexts is a long 3′ untranslated region (UTR) (Lykke-Andersen and Jensen, 2015). In support of the importance of this NMD-inducing feature, mRNAs encoded by *Upf2*-null blastocyst-upregulated genes had an average 3′ UTR length almost twice as long as mRNAs in general (Fig. S2E; Table S2). However, this was not the case for genes encoding mRNAs stabilized or both stabilized and upregulated in *Upf2-*null blastocysts (Fig. S2E; Table S2). This raises the possibility that 3′ UTR length is not a crucial determinant in eliciting NMD in blastocysts or that its role is complex, potentially involving mechanisms in addition to NMD.

Our final approach to identify bona fide NMD target mRNAs in blastocysts was to cross-reference with mouse mRNAs previously implicated as NMD targets. To achieve this, we established a list of putative and high-confidence mouse NMD target mRNAs defined in various biological contexts in the literature (Bao et al., 2015; Hurt et al., 2013; Mao et al., 2015; McIlwain et al., 2010; Mooney et al., 2017; Thoren et al., 2010; Weischenfeldt et al., 2012). Comparison with this list revealed a 35% overlap with genes upregulated in *Upf2-*null blastocysts, and 30% overlap with NMD-destabilized mRNAs (Table S2). This incomplete overlap is expected and does not negate the validity of the other putative NMD targets identified, as NMD target mRNAs are known to vary across cell types, in different biological contexts, and to respond to different NMD branch-specific factors (Fatscher et al., 2015; Huang and Wilkinson, 2012; Huang et al., 2011; Zetoune et al., 2008).

**Fig. 3.**
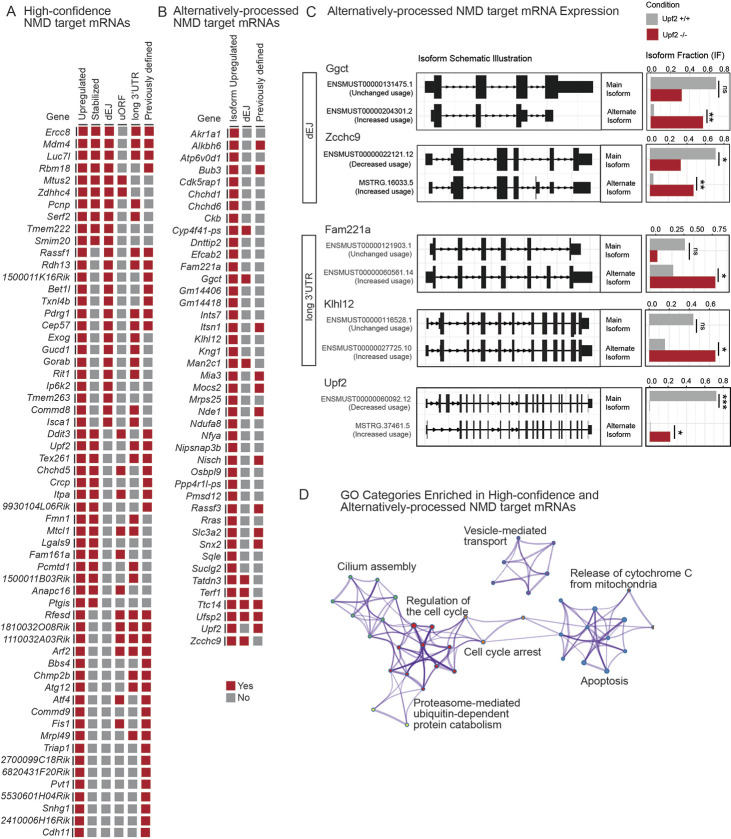
**Identification of high-confidence and alternatively-processed NMD target mRNAs in the mouse blastocyst.** (A,B) High-confidence (A) and alternatively-processed NMD targets (B), as defined in the text. Upregulated: mRNAs increased at the steady-state level in *Upf2-*null blastocysts. Stabilized: mRNAs for which turnover is decreased in *Upf2-*null blastocysts (Alkallas et al., 2017). Isoform upregulated: genes encoding at least one mRNA isoform significantly upregulated in *Upf2*-null blastocysts. dEJ, uORF and long 3′ UTR: NMD-inducing features. Previously defined: putative NMD target mRNAs defined in earlier studies (Table S2). (C) Isoform switch plots of selected genes, indicating the predominant and alternate isoform expressed in *Upf2*-null and control blastocysts. Bar graphs indicate the isoform fraction. Adjusted *P*-value (*Q*): ***Q<0.001; **Q<0.01; *Q<0.05; ns, not statistically significant (Bonferroni-adjusted *t*-test). (D) GO categories enriched in high-confidence and alternatively-processed NMD target mRNAs.

We integrated these three analyses to generate a list of 59 high-confidence NMD target mRNAs in the blastocyst (Fig. 3A; Table S2). To be on this list, the mRNA must be encoded by a gene upregulated in *Upf2-*null embryos and meet at least one of the following criteria: (1) be stabilized in *Upf2-*null embryos, (2) encode a transcript with a dEJ or (3) have been implicated as a NMD target in a previous published study, as defined above.

### Alternatively-processed NMD target mRNAs in the mouse blastocyst

Alternative processing of mRNAs commonly leads to production of isoforms targeted for decay by NMD (da Costa et al., 2017; Ge and Porse, 2014; Nasif et al., 2018). The most well-studied isoform-specific NMD targets are those produced by alternative splicing, which often shifts the reading frame, thereby generating a PTC (Ge and Porse, 2014; Lareau et al., 2007). It has been estimated that up to 30% of genes have alternatively-processed transcripts regulated by NMD (Lewis et al., 2003).

To identify alternatively-processed transcripts degraded by NMD, we used an established pipeline that detects isoform switches sensitive to NMD (Vitting-Seerup and Sandelin, 2017). We identified 66 genes encoding 84 mRNA isoforms undergoing a significant alteration in expression in *Upf2*-null versus control blastocysts (*Q*<0.1) (Fig. 3C; Fig. S3; Table S2). Among these is the *Upf2* gene itself, which loses two exons in *Upf2*-null blastocysts, explaining why an alternatively-processed isoform lacking these exons is upregulated (Fig. 3C). Because NMD target RNAs are upregulated after loss of an NMD factor, we focused our analysis on the 48 alternatively-processed isoforms (from 44 genes) upregulated in *Upf2*-null blastocysts (Fig. 3B; Table S2). None of these were identified in our steady-state analysis, underscoring the necessity of transcript-level analysis to identify regulated isoforms. Nine of the alternatively-processed isoforms (from eight genes) had a dEJ, identifying them as high-confidence NMD targets (Fig. 3B; Fig. S3). All nine of these dEJ-containing isoforms were upregulated by at least 2-fold in *Upf2*-null blastocysts (Fig. 3C; Fig. S3; Table S2). Four upregulated alternatively-processed isoforms had a significantly longer 3′ UTR than the major isoform(s) (Fig. 3C; Fig. S3; Table S2), and thus may be targeted for decay by this NMD-inducing feature (Lykke-Andersen and Jensen, 2015). Finally, 14 of the 48 upregulated alternatively-processed RNAs overlapped with genes previously implicated as NMD targets (Table S2).

In total, this analysis identified 22 high-confidence alternatively-processed NMD target mRNAs (from 19 genes), based on their being upregulated in *Upf2-*null blastocysts and having either a known NMD-inducing feature or corresponding to a previously identified target (Fig. 3B; Table S2). Most of the proteins predicted to be encoded by these alternatively-processed NMD target RNAs differ from the protein encoded by the most abundant mRNA isoform in wild-type blastocysts (Table S2).

### High-confidence NMD target mRNAs in the mouse blastocyst encode proteins promoting apoptosis and cell cycle progression

Together with the 22 high-confidence alternatively-processed NMD target mRNAs defined above, our analyses in total identified 78 genes encoding high-confidence NMD target mRNAs in the mouse blastocyst (Fig. 3; Fig. S2; Table S2). We evaluated this combined list of high-confidence NMD target mRNAs for enriched cellular processes. Four of the top six most highly enriched GO categories involve apoptosis or cell cycle progression (Fig. 3D), consistent with our above analyses of *Upf2*-regulated genes (Fig. 2A,C). The apoptosis-related categories include ‘positive regulation of apoptosis’, ‘intrinsic apoptotic signaling pathway’ and ‘release of cytochrome c from mitochondria’, the latter of which is widely recognized as a common step in response to multiple apoptotic stimuli (Garrido et al., 2006) (Fig. 3D). With respect to cell cycle regulation, enriched categories included ‘negative regulation of cell cycle’, ‘mitotic metaphase and anaphase’ and ‘chromosome localization’. These data further implicate NMD in influencing cell viability and proliferation in the early embryo.

### Upf2 is essential for the survival and progression of pluripotent cells

**Fig. 4.**
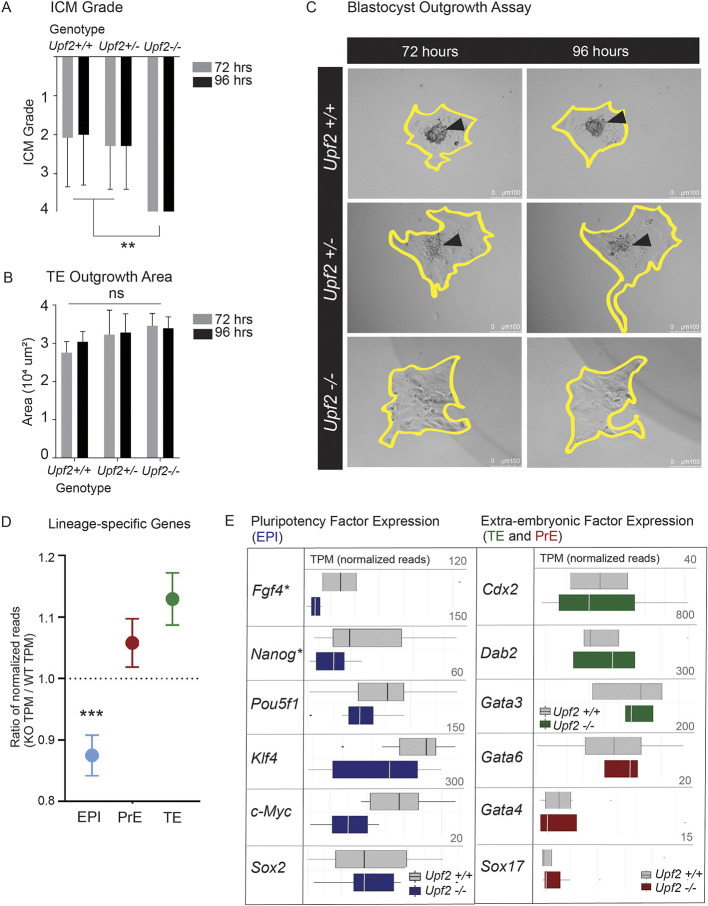
**Loss of *Upf2* is incompatible with ICM survival and leads to a reduction in pluripotent and epiblast-specific gene expression in the blastocyst.** (A) ICM grade after 72 and 96 h of outgrowth. The average grades for *Upf2^+/+^*, *Upf2^+/−^* and *Upf2^−/−^* ICMs were 2.1, 2.3 and 4.0 at 72 h, and 2.0, 2.3 and 4.0 at 96 h (**P<0.01; two-tailed Student's *t*-test). All *Upf2*-null embryos received an ICM grade of 4 (*n*=32). (B) TE outgrowth area (μm^2^) 72 and 96 h post-plating. Average expansion area is not significantly different for *Upf2*-null embryos and controls (72 h: *Upf2^+/+^*=27,556±2960; *Upf2^+/−^*=32,242±5919; *Upf2^−/−^*=34,596±2759; 96 h: *Upf2^+/+^*=30,373±2634; *Upf2^+/−^*=32,794±4551; *Upf2^−/−^*=33,952±2569) (*n*=32). (C) Representative brightfield images of blastocyst outgrowths at 72 and 96 h. TE expansion area is outlined in yellow. ICM is indicated by arrowheads. The ICM is clearly visible in *Upf2^+/+^* and *Upf2^+/−^* controls but absent in *Upf2^−/−^* embryos. (D) The mean ratio of normalized reads in *Upf2*-null relative to control blastocysts is plotted for epiblast (EPI)- (***P<0.0001, *n*=50 genes), PrE- (*P*=0.606, *n*=77 genes) (Ohnishi et al., 2013) or TE- (*P*=0.078, *n*=128 genes) specific genes (Blakeley et al., 2015). A ratio of less than one indicates lower expression in *Upf2*-null embryos. (E) The core epiblast factors *Fgf4* and *Nanog*, as well as the ‘Yamanaka factors’, *Pou5f1*, *Klf4*, *c-Myc* and *Sox2*, are downregulated in *Upf2*-null blastocysts (blue). Extra-embryonic markers in the TE (*Cdx2*, *Dab2*, *Gata3*; green) and PrE (*Gata6*, *Gata4*, *Sox17*; red) were unchanged. (*Q<0.1; Bonferroni-adjusted *t*-test). Data are mean±s.e.m. in A,B,D. In E, box plots show median values (middle bars) and interquartile ranges (boxes); whiskers indicate the maximum to minimum ranges.

The striking enrichment for genes involved in apoptosis and cell cycle regulation among NMD targets raised the possibility that loss of UPF2 causes embryonic lethality due to decreased proliferation and/or increased apoptotic cell death. To assess this, we performed an *in vitro* outgrowth assay, which assesses the initial stages of ICM proliferation and developmental progression, and measures the ability of TE to attach and initiate endometrial invasion (Armant et al., 1986). We graded ICM outgrowth (blinded to genotype) using the following standard numerical scale: 1, many cells forming a densely-packed epithelium; 2, many cells forming a loosely-packed epithelium; 3, few cells forming a loosely-packed epithelium; 4, no identifiable ICM (Gardner et al., 2000). Control ICMs had average scores of 2.0 (Upf2^+/+^) and 2.3 (Upf2^+/−^), whereas all *Upf2-*null ICMs had the lowest score – 4 – at both 72 and 96 h (Fig. 4A,C). This suggested that loss of *Upf2* prevents cell proliferation and/or progression, leading to rapid death of the ICM. In marked contrast to the complete blockade in ICM outgrowth, TE outgrowth was not detectably affected by *Upf2* loss. *Upf2*-null TE cells attached, differentiated into giant cells and expanded in a manner indistinguishable from controls (Fig. 4B,C). Indeed, the only cells remaining in *Upf2*-null blastocyst outgrowth cultures after both 72 and 96 h were sheets of trophoblast cells. We conclude that *Upf2* is required for the progression and survival of ICM cells, but is dispensable for the TE during peri-implantation development *in vitro.*

To further investigate this ICM-specific defect, we turned to our sequencing data to examine the expression of lineage-specific genes. We found that pluripotency marker genes were significantly downregulated (as a group) in *Upf2*-null blastocysts (Fig. 2B). As pluripotency genes are primarily expressed by epiblast cells, this raised the possibility of an epiblast-specific defect. To test this, we examined markers known to exhibit enriched expression in mouse epiblast, PrE or TE cells (Blakeley et al., 2015; Ohnishi et al., 2013). This analysis revealed that *Upf2-*null blastocysts expressed significantly lower levels of epiblast-specific genes compared with control embryos (*P*<0.0001; Fig. 4D,E). Among these were *Nanog*, *Fgf4*, *Pou5f1* (*Oct4*), *Sox2*, *Klf4* and *c-Myc* (*Myc*) (Chazaud and Yamanaka, 2016; Liu et al., 2008) (Fig. 4E). In support of an epiblast-specific defect, TE-specific (*P*=0.07) and PrE-specific (*P*=0.61) genes were not significantly altered in *Upf2*-null blastocysts (Fig. 4D,E).

**Fig. 5.**
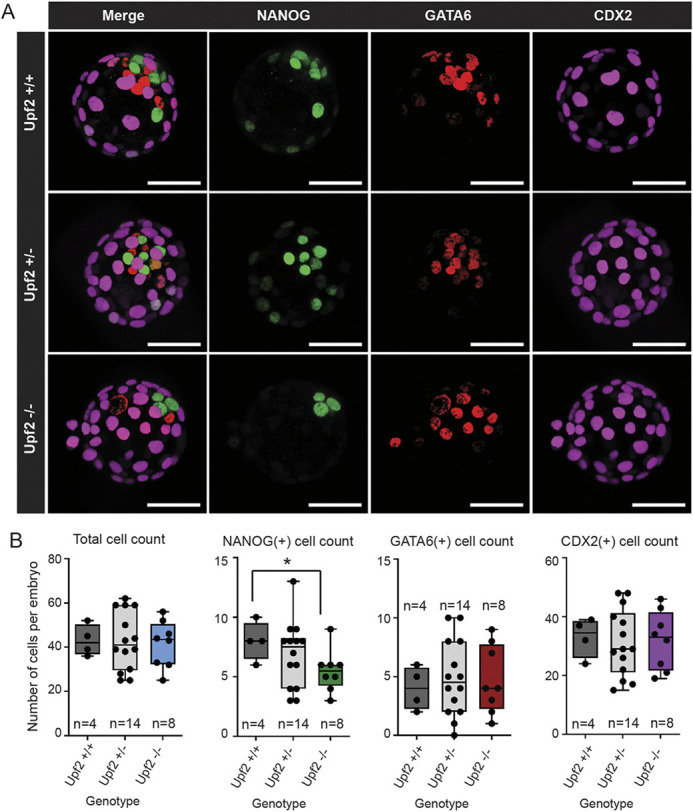
***Upf2-*null blastocysts contain fewer epiblast cells.** (A) Three-dimensional reconstructed volume images of *Upf2*-null and littermate control blastocysts, stained for NANOG (epiblast), GATA6 (PE) and CDX2 (TE). (B) Box and whisker plots of total cell counts per blastocyst. Total NANOG-positive cells per embryo was significantly lower in *Upf2*-null blastocysts relative to wild-type controls (*Upf2*^+/+^ versus *Upf2*^−/−^, *P=0.02, two-tailed Student's *t*-test). The number of GATA6-positive (*P*=0.33), CDX2-positive cells (*P*=0.43) and total cell number (DAPI-positive) (*P*=0.39) was unchanged. *n*=24 total blastocysts (*n*=8 *Upf2^−/−^*, *n*=14 *Upf2^+/−^*, *n*=4 Upf2^+/+^). Box plots show median values (middle bars) and interquartile ranges (boxes); whiskers indicate the maximum to minimum ranges; dots indicate individual data points. Scale bars: 50 μm.

Decreased expression of epiblast-specific genes could either result from a reduced number of epiblast cells or a reduction in expression of epiblast-specific genes. To distinguish between these possibilities, we stained blastocysts for the well-established markers NANOG (epiblast), GATA6 (PrE) and CDX2 (TE) (Wallingford et al., 2013), and quantified the staining by automated nuclear counting. This revealed a significantly lower number of NANOG-positive cells in *Upf2*-null blastocysts compared with littermate controls (*P*=0.02; Fig. 5; Fig. S4A). This effect was specific, as we did not observe a significant difference in the number of CDX2-positive (*P*=0.43) or GATA6-positive cells (*P*=0.33) (Fig. 5; Fig. S4). These findings likely do not represent a delay in the overall developmental stage of *Upf2*-null blastocysts given the total number of cells (*P*=0.39; Fig. 5B), total ICM cells (*P*=0.36; Fig. S4B) and the ICM/TE cell ratio (measure of blastocyst maturation/expansion) (*P*=0.48; Fig. S4C) were not significantly different.

To further assess this epiblast defect, we first attempted a number of approaches to directly assay the nature of the epiblast progression defect, including TUNEL analysis and immunofluorescence analysis of active caspase 3 expression in NANOG-positive cells during *in vitro* outgrowth. However, the very low number of NANOG-positive cells in *Upf2-*null blastocysts and their rapid depletion upon initiation of outgrowth prohibited making accurate quantitative comparisons. As an alternative, we asked whether *Upf2*-null blastocysts can generate embryonic stem cells (ESCs). In support of an epiblast defect, we were not able to derive stable *Upf2*-null ESC lines using conventional derivation conditions with serum and LIF-containing media (Bryja et al., 2006). Thus, despite *Upf2*-null blastocysts being present at the initiation of culture at a normal Mendelian ratio (Fig. 1E), only one *Upf2*-null blastocyst initiated growth (out of 28 lines from all genotypes), and it died with the first passage (Fig. S5A,B). In contrast, ESC lines were generated from littermate control blastocysts at a normal Mendelian ratio (Fig. S5A,B).

To probe the timing of the epiblast defect in *Upf2*-null blastocysts, we assessed whether these blastocysts could instead give rise to ESCs at a more naïve stage. It is well-established that naïve (or ‘ground state’) ESCs derived under 2-inhibitor (2i) (MEK inhibitor+GSK3 inhibitor) conditions (Czechanski et al., 2014) most closely resemble the early, unrestricted pre-implantation epiblast at E3.75-E4.5 (Boroviak and Nichols, 2014). In contrast, ‘primed ESCs’, which are derived under conventional conditions (with serum and LIF) instead represent post-implantation epiblast cells (E5.5-E6.5) about to enter gastrulation (Buecker et al., 2014; Hayashi et al., 2011). Using 2i-derivation conditions, we found that 12% (3 out of 25) of ESC lines generated from blastocysts obtained from *Upf2*-heterozygous matings were *Upf2*-null (Fig. S5A-C). Although 12% is less than the expected Mendelian ratio of 25%, our ability to derive several *Upf2*-null naïve ESCs indicates that UPF2 is not absolutely required for the naïve stage. Nonetheless, we found that *Upf2*-null naïve ESCs were abnormal, exhibiting slower cell expansion and increased apoptosis relative to littermate control lines grown in parallel (Fig. S5D,E). Together, these results – that UPF2 promotes the proliferation and survival of naïve ESCs and is essential for the progression and/or survival of ESCs cells to the primed state – further support that UPF2 has stage-specific roles in epiblast cells in the blastocyst.

### NMD magnitude is downregulated during peri-implantation development

**Fig. 6.**
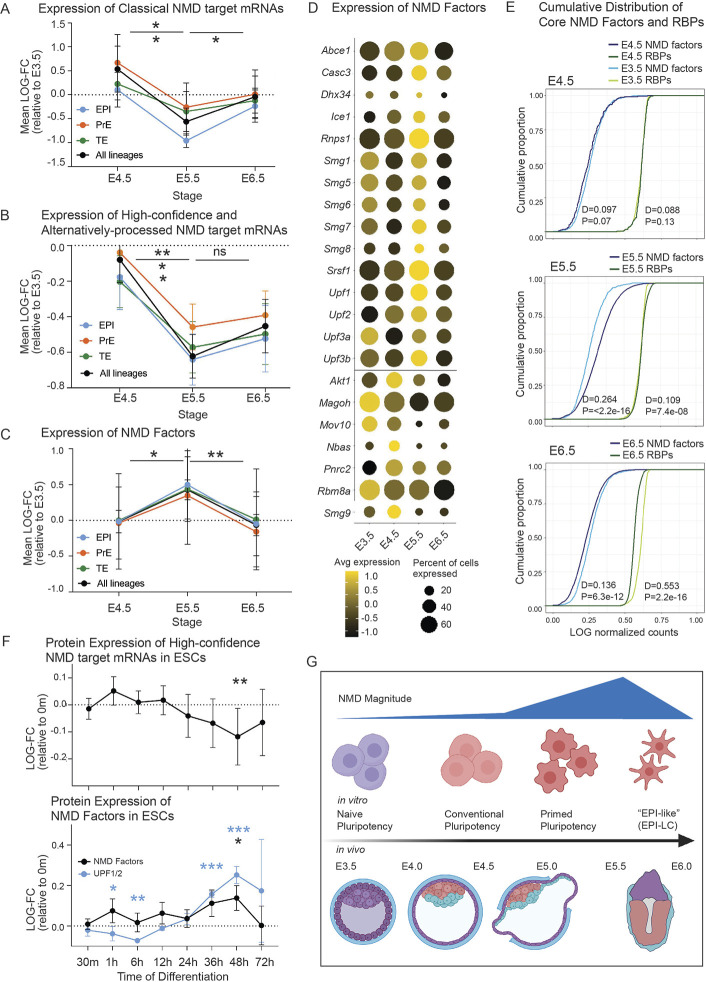
**NMD target mRNAs and NMD factors are concordantly regulated during peri-implantation development.** (A,B) The mean log (fold change) relative to E3.5 is plotted for (A) ‘classical’ NMD targets (Johnson et al., 2019) (*n*=6) and (B) high-confidence and alternatively-processed NMD targets (*n*=78), across peri-implantation development (Nowotschin et al., 2019). *P<0.05, **P<0.01; Student's *t*-test. (C) As in A and B, the mean log (fold change) relative to E3.5 is plotted for core NMD factors (*n*=11). *P<0.05, **P<0.01; Student's *t*-test. (D) Normalized expression of NMD factors, presented as a dot plot. Expression is graded by color and the proportion of cells expressing each gene at detectable levels is indicated by size. (E) Cumulative distribution of core NMD factor (*n*=11) and RBP (*n*=1066) expression, compared with E3.5. The Kolmogorov–Smirnov test was used to assess the difference between distributions via the Distance (D)-statistic. (F) Expression of NMD targets and factors at the protein level during differentiation of naïve mESCs (0 m) to form EpiLCs (∼72 h) (Yang et al., 2019). The log (fold change) of proteins encoded by high-confidence and alternatively-processed NMD targets (*n*=75) (top) and core NMD factors (*n*=14) (bottom) is plotted. NMD targets significantly decrease and NMD factors significantly increase after 48 h of differentiation, relative to 0 m (*P<0.05, **P<0.01, ***P<0.001; Student's *t*-test). Asterisk color corresponds to NMD factor group significantly affected. (G) Diagram of NMD magnitude during development *in vitro* and *in vivo.* NMD magnitude increases (NMD target expression decreases with a mirrored increase in NMD factors) during ESC priming *in vitro* and during implantation and epiblast expansion *in vivo.* Data are mean±s.e.m. in A-C,F.

One means by which NMD might influence developmental events is by undergoing a shift in magnitude, as this would, in turn, shift the stability of NMD target mRNAs and thereby alter the expression of their protein products. To investigate whether NMD magnitude is regulated during early embryo development, we examined the levels of well-established (‘classical’) NMD target mRNAs (Johnson et al., 2019) across peri-implantation development using a published single-cell RNA-seq dataset (Nowotschin et al., 2019). This revealed that these classical NMD target mRNAs are downregulated between E4.5 and E5.5 (Fig. 6A; Fig. S6A). To determine whether this regulation is cell-type specific, we segregated the data by lineage, revealing that NMD target mRNAs are most significantly downregulated in the epiblast (*P*=0.01) and not significantly regulated in the PrE and TE (*P*=0.13 and *P*=0.15, respectively) (Fig. 6A; Fig. S6A). This epiblast-specific shift in NMD target mRNAs at E5.5 appeared to be transient, as they were subsequently upregulated after E5.5 (Fig. 6A; *P*=0.04). We next examined the expression of the high-confidence NMD target mRNAs that we defined in blastocysts (Fig. 3). Just as we observed for the classical NMD targets, there was a significant downregulation of the high-confidence blastocyst NMD targets between E4.5 and E5.5 (Fig. 6B). In fact, almost 80% of these high-confidence NMD target RNAs were downregulated between E4.5 and E5.5 (Fig. S6B,C), demonstrating the pervasiveness of this regulation. The shift in high-confidence NMD target mRNAs occurred most notably in epiblast cells (Fig. 6B; Fig. S6C), just as observed for classical targets (Fig. 6A; Fig. S6A). The coordinated, stage- and lineage-specific downregulation of this large cohort of NMD target mRNAs is strong evidence for an upward shift in NMD magnitude between E4.5 and E5.5.

One mechanism by which NMD magnitude could be upregulated is through upregulation of NMD factors. To test this, we examined the temporal expression pattern of the 22 NMD factor genes listed in Fig. 6D (Chan et al., 2009; Chang et al., 2007; Kishor et al., 2019; Kurosaki et al., 2019; Longman et al., 2013; Palma et al., 2021; Popp and Maquat, 2013; Schweingruber et al., 2013). This analysis revealed significantly increased expression of these 22 NMD factor genes (as a group) between E4.5 and E5.5 (Fig. 6C). More than half of these NMD factor genes were upregulated (Fig. 6D, upper), including the core NMD factor genes (*Upf1*, *Upf2*, *Upf3b*, *Smg1*, *Smg5*, *Smg6* and *Smg7*), all of which encode proteins that drive key events in NMD (Chang et al., 2007; Popp and Maquat, 2013). Following upregulation at E5.5, these NMD factors were significantly downregulated (as a group) between E5.5 and E6.5 in all three cell lineages (Fig. 6C). As an independent approach, we plotted the cumulative distribution of NMD factor gene expression. To account for global expression changes that might occur as a result of rapid proliferation, we normalized against the expression distribution of mRNAs encoding RNA-binding proteins (RBPs). This analysis verified the transient upregulation of NMD factors at E5.5 (Fig. 6E; Fig. S6D,E). Lineage-specific analysis revealed that, although all three cell lineages exhibited upregulation of NMD factors at E5.5, the regulation was again most prominent in the epiblast (Fig. S6D,E). Together, these data demonstrate a transient upregulation of NMD factor genes at E5.5 – precisely the stage at which NMD target mRNAs are downregulated (Fig. 6A,B; Fig. S6A-C). This inverse regulation is consistent with a model in which upregulation of one or more NMD factors is responsible for the downregulation of NMD activity at E5.5.

To independently assess this shift in NMD magnitude, we turned to an ESC proteomics dataset (Yang et al., 2019), with time points that model epiblast differentiation from the blastocyst to peri-implantation stages, the same temporal period exhibiting defects in *Upf2-*null embryos*.* In particular, the ESCs used by this study are naïve ESCs [which resemble pre-implantation (E3.75-E4.5) epiblast cells] (Boroviak and Nichols, 2014) that were cultured to form epiblast-like cells (EpiLCs) [which most closely resemble post-implantation pre-gastrulation (E5.5-E6.5) epiblast cells] (Buecker et al., 2014; Hayashi et al., 2011). We examined the expression of proteins encoded by the high-confidence NMD target RNAs we identified (Fig. 3), and found that their temporal expression mirrored their expression at the RNA level. Specifically, these proteins exhibited significantly reduced expression during the progression from naïve ESCs to EpiLCs (Fig. 6F, top). NMD factors, as a group, exhibited an inverse pattern, as predicted (Fig. 6F, bottom). The core NMD factors, UPF1 and UPF2, exhibited a particularly robust increase in expression, with significant increases at *in vitro* time points that corresponded to ∼E5.5 *in vivo* (36 and 48 h) (Fig. 6F, bottom). We conclude that NMD magnitude is increased in the early embryo at E5.5, leading to destabilization of many NMD target mRNAs and a corresponding decrease in the expression of their encoded proteins (Fig. 6G).

## DISCUSSION

Work from numerous laboratories has shown that loss or inactivation of any of a number of NMD factors causes early embryo lethality in eukaryotes, extending from flies to mice (Alonso and Akam, 2003; Anastasaki et al., 2011; Casadio et al., 2014; Hwang and Maquat, 2011; Li et al., 2015; Longman et al., 2007; Mao et al., 2015; McIlwain et al., 2010; Medghalchi et al., 2001; Metzstein and Krasnow, 2006; Shum et al., 2016; Silver et al., 2010; Weischenfeldt et al., 2008; Wittkopp et al., 2009). These data raise the possibility that NMD has one or more key roles in early development, but the underlying mechanism has remained largely unexplored. Using NMD-deficient *Upf2*-null mice, we show that loss of NMD leads to stage- and lineage-specific defects, with molecular and phenotypic defects occurring earlier than previously recognized. Consistent with the notion that NMD is not essential for all cells, loss of UPF2 and NMD did not measurably impair overall developmental progression to the blastocyst stage, nor did UPF2 loss detectably affect most cell types in the early embryo (Fig. 1; Fig. S1). Instead, our data indicate that NMD is specifically crucial for epiblast cells.

Why are epiblast cells preferentially sensitive to loss of NMD? One possibility stems from the fact that epiblast cells are highly proliferative. During peri-implantation development, epiblast cells undergo massive expansion, with cell numbers increasing from ∼5-10 cells at E3.5 to ∼600-700 cells at E6.5 (Chuva de Sousa Lopes, 2004; Lewis and Rossant, 1982; Power and Tam, 1993; Snow, 1977), and failure to adequately expand epiblast cells in the blastocyst predicts poor developmental progression to E6.5 (Morris et al., 2012b). Consistent with the notion that epiblast cells uniquely require UPF2 because they are highly proliferative, several proliferative cell types have been shown to die in the absence of UPF2, including hematopoietic progenitors, spermatogonia and perinatal Sertoli cells (Bao et al., 2015, 2016; Weischenfeldt et al., 2008). As evidence for specificity, hematopoietic cell-specific conditional knockout of *Upf2* results in the loss of rapidly proliferating progenitors, but not terminally-differentiated macrophages (Weischenfeldt et al., 2008).

Support for our finding that *in vivo* epiblast progression is perturbed in *Upf2-*null embryos (Fig. 5; Fig. S4) is our inability to generate stable primed ESC lines from *Upf2-*null blastocysts (Fig. S5). This is consistent with past studies showing that primed ESCs could not be derived from mouse blastocysts with loss-of-function mutations in NMD factor genes (Hwang and Maquat, 2011; Li et al., 2015; Medghalchi et al., 2001; Weischenfeldt et al., 2008). We then took this one step further and asked whether we could instead derive less-advanced (naïve) ESC lines from *Upf2*-null blastocysts. We were successful in this endeavor but found that these naïve *Upf2*-null ESCs had a growth defect and an increased rate of apoptosis relative to control ESCs. Together, these data suggest that NMD is essential for maintaining the pluripotent state of cells. In support, previous studies have shown that NMD regulates pluripotency and differentiation decisions. For example, high NMD activity has been found to maintain the stem-like state of neural progenitors and ESCs (Bruno et al., 2011, Lou et al., 2016, 2014). In response to pro-neurogenic signals, neural progenitors downregulate NMD, which strongly promotes their differentiation (Bruno et al., 2011; Lou et al., 2014). In contrast, evidence suggests that NMD has a positive influence on the differentiation of other cell types, including hepatoblasts and mouse ESCs (Li et al., 2015; Thoren et al., 2010). In human ESCs, NMD has a dual role: high NMD activity drives mesoderm differentiation, whereas low NMD activity drives endoderm differentiation (Lou et al., 2016). Specific NMD factors can have differing roles; e.g. UPF3B promotes mouse neural stem cell differentiation (Jolly et al., 2013), whereas UPF1 inhibits neural progenitor differentiation (Lou et al., 2014).

A key question is whether our results with mice lacking UPF2 are generalizable to other NMD factors. Although previous studies knocking out NMD factor genes have not determined the precise timing of mouse embryo lethality, they point towards a common defect in the peri-implantation period (Hwang and Maquat, 2011; Li et al., 2015; Mao et al., 2015; McIlwain et al., 2010; Medghalchi et al., 2001; Shum et al., 2016; Weischenfeldt et al., 2008). In the case of SMG1, its loss results in smaller embryos with death by early post-implantation development (McIlwain et al., 2010). Similar to our phenotypic data, *Upf1*-null (Medghalchi et al., 2001) and *Smg6*-null blastocysts (Li et al., 2015) undergo regression after a short period of culture. Also similar, we have shown that *Upf3a*-null blastocysts display developmental defects with death in the peri-implantation period (Shum et al., 2016). Here, we traced the role of NMD in the peri-implantation window to the epiblast specifically and uncovered NMD target mRNAs and a stage-specific shift in NMD activity. An enticing hypothesis that unifies these findings is that the NMD pathway serves as a regulatory pathway within the epiblast around the time of implantation, allowing for shifts in cohorts of mRNAs that are crucial for this pluripotent cell type. In the future, it will be important to test this hypothesis by conducting rigorous analysis of mice lacking different NMD factors. Of note, it is possible that the phenotype observed in this study was affected by maternal UPF2 inherited from the oocyte. Unfortunately, we could not test this possibility, as the UPF2 antibody most widely used in the field (Lykke-Andersen et al., 2000) is unable to differentiate between endogenous UPF2 and the truncated UPF2 produced by the *Upf2-*null mutant. However, if true, maternal UPF2 would be expected to mask and/or reduce defects in the embryo, suggesting that the phenotype of *Upf2*-null mice in this study might be an underestimate of the early embryonic functions of UPF2.

Another interesting future question is whether NMD has conserved roles in early embryo development that extend beyond mice. In zebrafish, knockdown of any of a number of different NMD factors causes patterning perturbations and reduced viability (Anastasaki et al., 2011; Wittkopp et al., 2009). It is likely that human embryonic development also depends on NMD, as there have been no reports of homozygous mutations in core NMD genes in humans, with the exception of UPF3B, which is not essential for NMD (Jaffrey and Wilkinson, 2018). In *Drosophila*, *Upf1* and *Upf2* are required for larval viability, in part because NMD confers a competitive growth advantage to fly embryonic cells (Metzstein and Krasnow, 2006). Given that NMD is known to drive the proliferation of several mammalian cell types (Azzalin and Lingner, 2006; Gehen et al., 2008; Lou et al., 2014; Lykke-Andersen and Jensen, 2015; Weischenfeldt et al., 2008), it is tempting to speculate that a conserved function of NMD is to drive the expansion of specific cell populations during early development. The role of NMD in *Caenorhabditis elegans* appears to be more complex, as knockdown of the NMD genes *Nbas* and *Dhx34* causes severe developmental defects (Longman et al., 2007), whereas complete knockout of other NMD genes, including core NMD genes, only causes male bursa and hermaphrodite vulva defects (Pulak and Anderson, 1993).

Towards understanding the molecular mechanisms by which NMD functions in early development, we identified high-confidence NMD target mRNAs in blastocysts using several approaches (Fig. 3; Figs S2,S3). Many of these targets encode proteins involved in apoptosis (Fig. 3D), raising the possibility that, through downregulation of pro-apoptotic factor mRNAs, NMD directly promotes cell survival. One of the mouse blastocyst NMD target mRNAs we identified encodes GADD45A, a pro-apoptotic signaling factor (Tamura et al., 2012). The *D. melanogaster* ortholog *GADD45* must be downregulated by NMD to permit *Drosophila* cell survival and embryo viability (Nelson et al., 2016). This raises the intriguing possibility that decay of *Gadd45a* mRNA by NMD in the mouse embryo serves the same purpose. We identified a number of other pro-apoptotic factor mRNAs targeted for decay by NMD in the mouse blastocyst. These include *Fis1* and *Ggct*, which encode proteins that induce cytochrome c release from mitochondria (Masuda et al., 2006; Yu et al., 2005), *Smim20*, which encodes a key component of a protein complex that regulates cytochrome c oxidase assembly (Dennerlein et al., 2015), and *Mdm4*, which encodes a pro-apoptotic protein that migrates to the mitochondria in response to cell stress, leading to cytochrome c release (Mancini et al., 2009). As further support for the notion that NMD targets pro-apoptotic mRNAs, McIlwain et al. found that knockout of *Smg1* upregulates many mRNAs encoding proteins with roles in cell death in murine embryo fibroblasts derived from post-implantation embryos (McIlwain et al., 2010). Together, these findings support a model in which mouse blastocysts are in a precarious apoptotic-sensitive state (Azzalin and Lingner, 2006). NMD prevents their death by apoptosis, but if NMD is perturbed, this unleashes pro-apoptotic factors, leading to the *Upf2-*null blastocyst phenotype we defined: ICM outgrowth failure and reduced epiblast cells.

Many blastocyst NMD target mRNAs also encode proteins involved in the cell cycle and cell proliferation. Interestingly, a number of the mRNAs in this class encode negative cell-cycle factors, raising the possibility that these mRNAs must be degraded by NMD to permit rapid cell growth. Consistent with this idea, we previously showed that several mRNAs encoding proliferation inhibitors are targeted for decay by NMD in mouse P19 embryonal carcinoma stem cells (Lou et al., 2014). Although the functional relevance of these targets was not determined, we provided evidence – through both loss- and gain-of-function studies – that NMD promotes the proliferation of P19 cells (Lou et al., 2014). Further support for the notion that NMD promotes cell growth comes from loss-of-function studies in other mammalian cell lines (Azzalin and Lingner, 2006; Gehen et al., 2008; Lykke-Andersen and Jensen, 2015; Weischenfeldt et al., 2008) as well as *Drosophila* (Avery et al., 2011; Rehwinkel et al., 2005). An interesting class of blastocyst NMD target mRNAs that we identified are those encoding proteins involved in both cell-cycle arrest and cell death (e.g. DDIT3 and ATF4; Gardner, 2008; Ohoka et al., 2005; Yamaguchi and Wang, 2004), making them particularly attractive candidates to require suppression by NMD for successful embryo progression.

We also identified a number of alternatively-spliced mRNAs targeted for decay by NMD in the blastocyst. This is not surprising, as alternative splicing shifts reading frame one-third of the time, which almost invariably leads to the generation of a PTC. Such out-of-frame NMD target mRNAs are often generated by regulated alternative RNA splicing, a phenomenon referred to as ‘alternative splicing (AS)-NMD’ (da Costa et al., 2017; Ge and Porse, 2014; Lareau et al., 2007). In this mechanism, regulated splicing and NMD converge to act as an ‘on-off’ switch. The switch is in the ‘on state’ when the normally spliced isoform is produced, as it is insensitive to NMD. The switch is in the ‘off state’ when an appropriate cellular cue is received that switches RNA splicing to generate the alternatively-spliced NMD-sensitive isoform. Because the alternatively-spliced mRNA is rapidly degraded by NMD, it can only produce small amounts of protein. Facilitating this ‘off state’, the alternative splicing event often generates a non-functional protein. In support of AS-NMD regulation serving this role in the mouse blastocyst, we identified many alternatively-spliced blastocyst NMD target mRNAs encoding proteins that are likely to be non-functional (Fig. 3B,C). However, some of these mRNAs could instead encode novel functional proteins. Those missing a single domain are candidates to act as dominant-negative inhibitors, and those encoding new amino acids may have a different function than that encoded by the dominant mRNA isoform (Backwell and Marsh, 2022).

Transcription factors are regulated during development to shift the expression of their gene targets. By analogy, evidence suggests that the NMD pathway is regulated to achieve the same goal (Huang and Wilkinson, 2012; Karam et al., 2013). Here, we provide several lines of evidence that NMD magnitude is transiently increased in epiblast cells during peri-implantation development at approximately E5.5 (Fig. 6; Fig. S6). Consistent with this *in vivo* observation, we obtained evidence that NMD magnitude is increased when ESCs progress to the corresponding stage *in vitro* (Fig. 6F). This transient upregulation of NMD would be predicted to increase the decay of NMD target mRNAs. Indeed, we found that ∼80% of the blastocyst NMD target mRNAs we identified are downregulated at E5.5 *in vivo* (Fig. S6B) and at the corresponding stage of ESC maturation *in vitro* (Fig. 6F,G). We suggest that the lineage-specific downregulation of one or more of these mRNAs in response to increased NMD magnitude at this time point is essential for subsequent events during embryo development. Although we do not know the mechanism responsible for this transient increase in NMD magnitude, we found that a large cohort of NMD factors are transiently upregulated at the same embryonic time point, consistent with their upregulation being responsible for the increased NMD magnitude (Fig. 6; Fig. S6). The mechanism responsible for this coordinated upregulation of many NMD factors is an interesting future question; one possibility is that these NMD factor genes are regulated by a common transcription factor responsive to a stage-specific developmental cue. Future studies are necessary to tease apart how coordinated regulation on this scale is mediated to influence development.

We conclude that the NMD factor UPF2 influences stage- and lineage-specific cellular and molecular events during the peri-implantation period that drive progression of epiblast cells in the blastocyst and ultimately permit survival of the early embryo.

## MATERIALS AND METHODS

### Generation of global heterozygous mice

Global knockout (heterozygous) mice were generated by crossing mice expressing Cre recombinase driven by the *E2a* promoter in the early preimplantation embryo to a previously published *Upf2*-floxed mouse in the C57BL/6 background (Weischenfeldt et al., 2008). The *Upf2*-floxed construct was designed to delete exons 2 and 3, and although a truncated protein is still generated, it has been shown to lack NMD activity (Weischenfeldt et al., 2008). Global heterozygous males and females were bred to generate mice for experiments, and all experiments herein were conducted on embryos obtained from global *Upf2*-heterozygous parental crosses. For each experiment detailed below, the total number and age of mice used are clearly stated. Mice were genotyped using polymerase chain reaction (PCR) (primers used for genotyping are provided in Table S1). The mice colonies were maintained in agreement with protocols approved by the Institutional Animal Care and Use Committee at the University of California, San Diego (CA, USA).

### Genotyping and DNA PCR

Ear tags were taken from mice and DNA was extracted for PCR. Lysed ear tags were added to 0.2 μg/ml Proteinase K in DirectPCR lysis buffer (Viagen, 103-T), and then heated at 55°C for 3-4 h and then 85°C for 30 min to denature lysozymes in the buffer. PCR reactions were conducted in 15 μl volume reactions with 1 μl lysed mouse DNA, 10.8 μl nuclease-free water, 0.6 μl dNTP mixture (BioPioneer, MDM-4), 0.6 μl forward and reverse primer mixture (10 μM), 1.5 μl 10× buffer (Denville, CB3702-7) and 0.5 μl Taq polymerase (Denville, CB4050-2). We used 1.2% agarose gel with ethidium bromide for visualization of gene bands.

Genotyping of single embryos was performed using a custom low-input protocol. Early post-implantation embryos were carefully dissected out of the maternal decidua and washed in 2.5% pancreatin/0.5% trypsin in Tyrode Ringers Solution to remove external membranes and any resulting maternal tissue contamination. DNA was extracted for PCR by incubation in 10 μl Blastocyst Lysis Buffer (100 mM Tris-HCl, pH 8.3, 100 mM KCl, 0.02% gelatin, 0.45% Tween 20, 20 mg/ml Proteinase K) for 30 min at 55°C followed by incubation at 95°C for 10 min to denature active lysozymes. Genotyping PCR reactions were performed using a high sensitivity Taq (PrimeSTAR HS DNA Polymerase, Takara R010A). Reactions were conducted in 20 μl volume reactions with 1-3 μl embryo DNA (1 μl for post-implantation embryos, 3 μl for preimplantation embryos), 1.6 μl dNTP mixture, 2 μl primer mixture (10 μM) and 0.2 μl PrimeSTAR Taq, with nuclease-free water to total volume. Preimplantation embryos required 38 cycles for accurate visualization of bands on a 1.2% agarose gel with ethidium bromide.

### Isolation and primary culture of zygotes and isolation of blastocysts

For experiments involving the isolation and primary culture of zygotes, female mice of age 4-8 weeks were stimulated with intraperitoneal injection of 5 IU equine chorionic gonadotropin (eCG; Lee BioSolutions, 493-10) followed by intraperitoneal injection of 5 IU human chorionic gonadotropin (hCG; Sigma-Aldrich, C1063) 46-48 h later. Immediately after hCG injection, female mice were placed in the same cage as a stud male of known fertility for mating. The contents of the ampulla were collected only from females with a visible plug 18 h after hCG injection. Ampulla contents were evaluated for the presence of zygotes by microscopy following a brief digestion in 0.1 mg/ml hyaluronidase (Sigma-Aldrich, H4272) dissolved in M2 media (Sigma-Aldrich, M7176). Zygotes were washed four times in M2 before being transferred to M16 (Sigma-Aldrich, M7292) for culture. Embryos were cultured in 20 μl drops of M16, overlaid with mineral oil, 20 embryos per drop. For blastocyst experiments, zygotes were cultured at 37°C, and were isolated 90-94 h post-hCG, 100-104 h post-hCG and 110-114 h post-hCG, for early-, mid- and late-blastocyst stages, respectively. For some experiments, blastocysts were isolated from the uterine tubes of females that had been superovulated with eCG and hCG, as detailed above, and mated. E3.5 blastocysts were isolated ∼96 h post-hCG injection, from females with a visible plug 18 h after hCG injection. The uterine tubes were dissected apart and M2 media was flushed through, using an insulin syringe, to expel the embryos. Statistical analysis of comparisons between genotypes was conducted using two-tailed Student's *t*-tests.

### Isolation of early post-implantation embryos

To obtain early post-implantation embryo counts, timed matings were set up for male and female *Upf2-*heterozygous mice. Female mice were 8-12 weeks of age, and male mice were stud males of known fertility. Females were examined for the presence of vaginal plugs following mating. Only females with observable plugs were used for post-implantation embryo dissections. On E5.5 and E6.5, female mice were injected with 0.1 ml of a 1% Chicago Blue dye solution through the tail vein using a 1 ml syringe fitted with a 27-gauge needle. Mice were sacrificed approximately 3 min after the dye injection and the uterine horns were carefully removed. Implantation sites could then be visualized by the blue dye. Each embryo was separated by cutting between implantation sites along the uterine horn. The muscular uterine myometrium was peeled back to expose the decidua, and the decidua was carefully dissected back to reveal each embryo. Reichert's membrane, if still attached, was removed by washing in 2.5% pancreatin/0.5% trypsin in Tyrode Ringers Solution, as well as careful dissection, if necessary (Govindasamy and Bedzhov, 2019). Statistical analysis of comparisons between genotypes was conducted using two-tailed Student's *t*-tests.

### Outgrowth assay

Blastocysts were obtained from 4- to 8-week-old female *Upf2*-heterozygous mice, as per above. To assay blastocyst attachment and outgrowth, blastocysts were individually plated in a single well of a 24-well gelatin-coated (0.1% gelatin) plate in α-MEM containing 1% fetal bovine serum and 1% penicillin/streptomycin. After 72 and 96 h of incubation at 37°C, images were taken for further analysis. After 96 h, blastocyst outgrowths were washed twice with PBS and individually picked for genotyping. Statistical analysis of comparisons between genotypes was conducted using two-tailed Student's *t*-tests.

### Derivation and culture of mouse ESCs

Mouse ESCs were derived by *in vitro* expansion of E3.5 blastocysts, which were obtained by breeding *Upf2-*heterozygous mice. Briefly, following the previously described protocol for isolation of mouse ESC from non-permissive lines, blastocysts were cultured on irradiated mouse embryonic feeders (MEFs) (Thermo Fisher Scientific, A34181) in a mixture of DMEM/F-12 and Neurobasal media with N2 and B27 supplements. The media was further supplemented with MEK (1 μM, PD325901) and GSK (3 μM, CHIR99021) inhibitors along with LIF (EMD Millipore, ESG1106) (2i-LIF) and 2% knockout serum replacement. Once blastocysts attached to the feeder layer, half media changes were carried out every 2 days. Cells were maintained in 2i-LIF media for 10-12 days. Large clusters of outgrown cells were then split and maintained either in 2i-LIF or in serum containing mouse ESC media containing KnockOut DMEM (Thermo Fisher Scientific, 10829018), 20% ESC-qualified fetal bovine serum (Thermo Fisher Scientific, 10439024), L-glutamine and supplemented with non-essential amino acids and 1000 U/ml of recombinant mouse Lif (EMD Millipore, ESG1106). Statistical analysis of comparisons between genotypes was conducted using two-tailed Student's *t*-tests.

### Immunofluorescence

Blastocysts were isolated from *Upf2-*heterozygous females aged 4-8 weeks as per above, following superovulation. The zona pellucida was removed in Acid Tyrode's solution, followed by three washes in PBS with 0.1% bovine serum albumin (BSA). Embryos were fixed with 4% paraformaldehyde in PBS for 1 h at room temperature followed by three washes in PBS with 0.1% BSA and 0.1% Tween (PBST). The embryos were then permeabilized by incubation with 1% Triton X-100 in PBS for 1 h at room temperature. After permeabilization, the embryos were again washed three times in PBST and blocked with 4% normal donkey serum in PBST for 2 h at room temperature. The embryos were incubated with primary antibodies overnight at 4°C. All washes and incubations were performed with gentle agitation. Anti-CDX2 antibody (Thermo Fisher Scientific, MA5-14494) was used at a dilution of 1:500, anti-NANOG antibody (Invitrogen, 14-5761-80) was used at a dilution of 1:500 and anti-GATA6 (R&D Systems, AF1700) was used at a dilution of 1:500. After removing the primary antibody, the embryos were washed three times in PBST and then incubated with Alexa Fluor 647 conjugated donkey anti-rabbit IgG (H+L) secondary antibody (Thermo Fisher Scientific, CA-31573), Alexa Fluor 488 conjugated donkey anti-rat IgG (H+L) Highly Cross-Adsorbed secondary antibody (Thermo Fisher Scientific, A-21208) and Alexa Fluor 546 conjugated donkey anti-goat IgG (H+L) Highly Cross-Adsorbed secondary antibody (Thermo Fisher Scientific, A-11056) (all diluted 1:1000) for 2 h at room temperature. The embryos were then washed three times with PBST and incubated with DAPI (Novus Biologicals, NBP2-31156) at a dilution of 1:1000 for 30 min. Embryos were washed with PBST three more times before being placed in individual 1 μl drops of PBS in a Nunc glass bottom dish (Thermo Fisher Scientific, 150682) and overlaid with mineral oil for imaging. Statistical analysis of comparisons between genotypes was conducted using two-tailed Student's *t*-tests.

To detect cell death, cells were co-stained with Annexin V and Propidium Iodide, and flow cytometry was used for analysis and quantification. Annexin V provides a very sensitive method for detecting cellular apoptosis and Propidium Iodide detects cells in late apoptosis or necrosis.

### Imaging methods

Embryos were imaged using a Nikon A1R confocal with a four-line LUN-V laser engine and DU4 detector, mounted on a Nikon Ti2 using a S Fluor 40×0.9 NA objective. Images were acquired in resonant mode with bidirectional scanning and 4× line averaging, and 0.575 µm steps were used to collect *z*-stacks of the entire embryo. The lasers used were 405 nm (7% laser power), 488 nm (5% laser power), 561 nm (3% laser power) and 640 nm (3% laser power). To avoid cross-talk between channels, *z*-stacks were acquired of the DAPI and Alexa Fluor 568 channels first, and the Alexa Fluor 488 and Alexa Fluor 647 channels were acquired subsequently.

### Automated nuclei/cell counting

To count nuclei positive for the three markers of interest, we created an automated image processing and counting routine using the General Analysis 3 tool within NIS Elements 5.11. For pre-processing, we applied local contrast, smoothing and a rolling ball filter before a threshold was applied to generate a binary layer. The binary was then subsequently eroded and dilated, cleaned, smoothed, and touching binaries were separated and filtered for size. The resulting binaries were then counted and the records were pooled. For each image stack, the routine was validated and adjusted manually.

### cDNA synthesis and library preparation of individual blastocysts

In total, 19 early-stage (E3.25) blastocysts (eight *Upf2^−/−^*, 11 *Upf2^+/+^*) were subjected to mRNA-sequencing. RNA isolation and cDNA synthesis were carried out using the SMARTer^®^ Ultra^®^ Low Input RNA Kit for Sequencing – v4 (Clontech, 634888). Embryos were genotyped after cDNA synthesis (before library preparation) by PCR (Table S1). Genotyping of cDNA was performed using custom primers (Table S1). In brief, as loxP sites surround exons 2 and 3 of the *Upf2* gene, the wild-type locus was detected using forward and reverse primers in exons 1 and 2, respectively; the knockout locus was detected using forward and reverse primers in exons 1 and 4, respectively. Genotype was confirmed via read alignment at *Upf2* exons 2 and 3 (Fig. S2A). Libraries were generated from the resulting cDNA (0.2 ng/μl per sample) of wild-type and *Upf2-*null embryos using the Nextera XT DNA library preparation kit (Illumina, FC-131-1024) as previously described (Mora-Castilla et al., 2016). Indexed sequence libraries were pooled for multiplexing, normalized by MiSeq read number and paired-end sequencing was performed on a HiSeq 4000.

### Analysis of mRNA-sequencing data

Reads were mapped via STAR (2.5.2a) (Dobin and Gingeras, 2015; Dobin et al., 2013) after trimming for adapter sequences. Samtools (Li et al., 2009) was used to process sam files, as well as to sort and remove PCR duplicates of bam files. Counts for each gene were quantified using the Subread package FeatureCounts using the gene level quantification in paired-end mode (release 1.5.2) (Liao et al., 2014) and annotated using the Ensembl GRCm38 genome. Reads were filtered such that genes without at least one sample with at least ten raw reads were removed from the analysis. Count data was normalized using the Bioconductor package edgeR (McCarthy et al., 2012; Robinson et al., 2010) and transcripts per million (TPM) counts were calculated. Differential expression was calculated using DESeq2 (Love et al., 2014) based on a model using the negative binomial distribution. Genes with an adjusted *P*-value less than 0.05 were considered differentially expressed unless otherwise noted, at which time an adjusted *P*-value of less than 0.1 was used. Heatmaps were constructed using the heatmap.2 function in ggplot2 (Wickham, 2016). Principal components were calculated with the prcomp function. All box plots, scatter plots, principal component analysis plots and heatmaps were constructed using ggplot2. All of these analyses and plot constructions were performed with RStudio, R (v3.2.2 and v3.4.0). GO analysis was performed using the online Metascape platform (Tripathi, 2015) with the mouse reference as the background gene set, and gene enrichment networks were visualized with Cytoscape (Shannon et al., 2003).

To identify putative NMD features, we used a previously published in-house Python script (Shum et al., 2015). Only Ensembl transcripts with a detectable 5′ UTR and 3′ UTR were considered for analysis. NMD features were calculated at the transcript level, and then collapsed to the gene level such that if a gene encoded any transcript that had an NMD feature, it was called positive. Statistical analysis was conducted using a Student's *t*-test, unless otherwise stated. Bonferroni-adjusted values for multiple comparisons was applied, where appropriate.

### Inference of mRNA stability from steady-state RNA-sequencing data

To infer mRNA stability from steady-state sequencing data, we employed the use of REMBRANDTS (Removing Bias from RNA-seq Analysis of Differential Transcript Stability) (Alkallas et al., 2017), which internally uses DESeq to obtain estimates of pre-mRNA and mature mRNA abundance and estimates a gene-specific bias function. The stringency parameter used for these analyses was 0.99, which is the most stringent value possible for this parameter.

### Alternative-splicing analysis

From the resulting sequencing files (FASTQ files) we removed adapter contamination and low quality base pairs with Trimmomatic v 0.32 (Bolger et al., 2014) using ILLUMINACLIP:fastqc.fa HEADCROP:15 LEADING:22 SLIDINGWINDOW:4:22 MINLEN:25, where the fastqc.fa file is a fasta file containing all the adapters that are distributed with FastQC v0.11.2. Quality of all libraries was checked with the FastQC tool (Babraham Institute, Cambridge, UK) both before and after trimming. Both paired and non-paired surviving reads were mapped as unstranded reads to the mouse reference genome (mm10) using Hisat2 v2.0.1-beta (Kim et al., 2015) with known splice sites from GencodeM12 (Harrow et al., 2012) added and Hisat2 was set to annotate properly paired reads as those with a minimum insert size in the interval from 0 to 1000 nucleotides.

We used StringTie v1.3.3b (Pertea et al., 2015) to predict potential novel transcripts in each sample, guided by the GencodeM12 (Harrow et al., 2012) transcript database, specifying mitochondrial genes to be ignored and a minimum isoform fraction of 0.1. The resulting individual transcriptomes were merged to create a combined transcriptome of all transcripts observed in any sample by using StringTie with the --merge as well as enabling the option of keeping introns. Afterwards, StringTie was used to quantify the combined transcriptome in each individual sample by using the -e -B parameters.

To systematically analyze the changes in the transcriptomes between wild type and knockout, we used IsoformSwitchAnalyzeR (Vitting-Seerup and Sandelin, 2017) v1.9.3 with minor modifications to the standard workflow. Specifically, the StringTie quantifications were imported using tximport (Soneson et al., 2015) v1.14 via the importIsoformExpression() function specifying readLength=86 (which is the average length after trimming). We removed isoforms expressed less than 0.1 TPM and not contributing at least 5% of the total parent gene expression. After this filtering, only genes with at least two transcripts were kept. The statistical analysis of differential used transcripts was carried out using DEXSeq (Anders et al., 2012) v1.32.0, using the isoformSwitchTestDEXSeq() function specifying reduceFurtherToGenesWithConsequencePotential=FALSE. ORFs, NMD-senstive isoforms and alternative splicing were identified and analyzed by IsoformSwitchAnalyzeR as described as in Vitting-Seerup and Sandelin (2017) and Vitting-Seerup et al. (2014). To describe the transcriptional changes we used the IsoformSwitchAnalyzeRs analyzeSwitchConsequences() function to analyze isoform switches for the following consequences (differences): intron retention, NMD, alternative transcription start and termination sites, changes in the last exon, changes in isoform length and exon number.

### Analysis of published single-cell embryo dataset

To assess expression dynamics of NMD factor and target RNAs during pre-, peri- and post-implantation development, we obtained Seurat-normalized counts from a large published single-cell mouse embryo dataset (Nowotschin et al., 2019; Stuart et al., 2019). Logarithmic fold change was calculated manually, using E3.5 as the control comparison, and data were plotted as mean (±s.e.m.) or summed (magnitude) Log (fold change). Dot plots were created using Seurat v3 (Stuart et al., 2019). Cumulative distribution frequency (CDF) plots were constructed via ggplot2 (Wickham, 2016), using the stat_ecdf() function. The Kolmogorov–Smirnov test was used to assess the difference between distributions. Distance (D)-statistics were calculated and represent a measure of the distance between two CDF plots. For NMD factor and RBP analysis, the Differential D-statistic was calculated by dividing the D-statistic for NMD factors by the D-statistic for RBPs. For NMD targets, only those that were significantly differentially expressed (*Q*<0.05) were included in resultant analysis and plots. Statistical analysis was conducted using Student's *t*-test, unless otherwise stated. Bonferroni-adjusted values for multiple comparisons was applied, where appropriate.

## Supplementary Material

10.1242/develop.200764_sup1 Supplementary information
