# Supplementary material for: Progression of the pluripotent epiblast depends upon the NMD factor UPF2
Source: Development. 2022 Nov 7;149(21):dev200764. doi: 10.1242/dev.200764 (PMC9687065; doi:10.1242/dev.200764)
Supplement: Supplementary information [file develop-149-200764-s1.pdf]

Figure S1

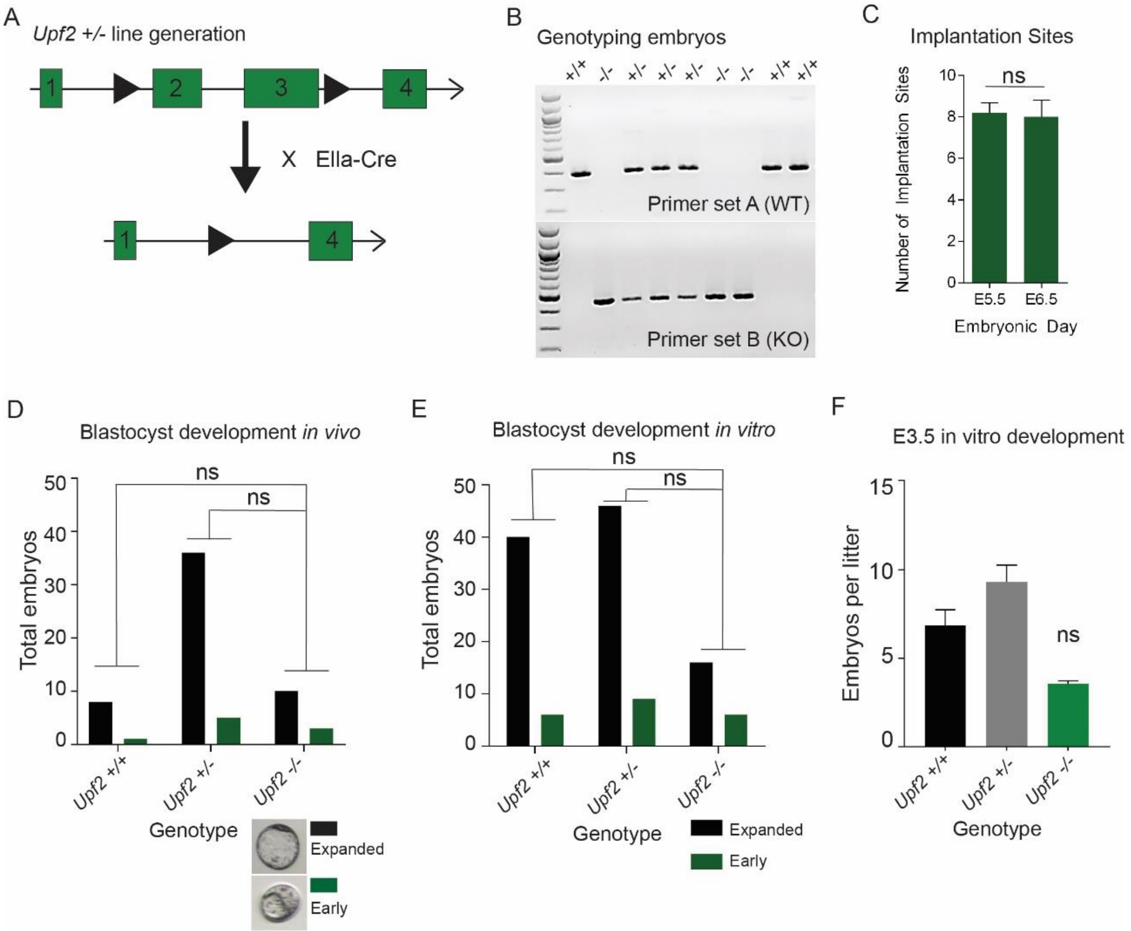

Fig. S1. Generation of global Upf2$^{+/-}$ mice and additional characterization of *Upf2*-null embryo phenotype. (A) Schematic for generation of global *Upf2*$^{+/-}$ knockout mice. *Upf2*$^{Fl/Fl}$ mice (obtained from Bo Porse laboratory), with loxP sites (black triangles) surrounding exons 2 and 3, were crossed with early embryo Ella-Cre mice to generate a global knock-out. **(B)** Genotyping individual embryos, as detailed in Experimental Procedures. Primer Set A (top) detects wild-type locus and Primer Set B (bottom) detects knock-out locus (sequences found in Table S1). **(C)** Total number of implantation sites detected for early post-implantation isolations for *Upf2*$^{+/-}$ crossings. E5.5 and E6.5 embryo isolations produced an average of 8.2 (n=5 breeding pairs, 34 embryos) and 8.0 (n=4 breeding pairs, 26 embryos) implantation sites, respectively; P=0.77. **(D-E)** Blastocyst development **(D)** *in vivo* and **(E)** *in vitro***.** Blastocysts were flushed from uterine horns of superovulated *Upf2*$^{+/-}$ females (mated with *Upf2*$^{+/-}$ males) at E3.5, or isolated as zygotes and cultured *in vitro*. Blastocysts were categorized as early stage or expanded, as demonstrated by brightfield images. *Upf2*-null blastocysts developed at a rate not significantly different than *Upf2*$^{+/+}$ controls and *Upf2*$^{+/-}$ controls (proportion of early to expanded, *in vivo Upf2*$^{+/-}$ *vs Upf2*$^{-/-}$, P=0.48 (n=63 embryos, 7 breeding pairs); *in vitro Upf2*$^{+/-}$ *vs Upf2*$^{-/-}$, P=0.16). **(F)** Zygotes were isolated from *Upf2* $^{+/-}$ females (superovulated and bred with *Upf2*$^{+/-}$ males) and cultured *in vitro* to the blastocyst stage. Data is presented as embryos per litter. *Upf2*-null blastocysts were present at the expected Mendelian ratio (11 breeding pairs, 123 embryos, P=0.09, T-test *Upf2*$^{+/+}$ *vs Upf2*$^{-/-}$).

Development • Supplementary information

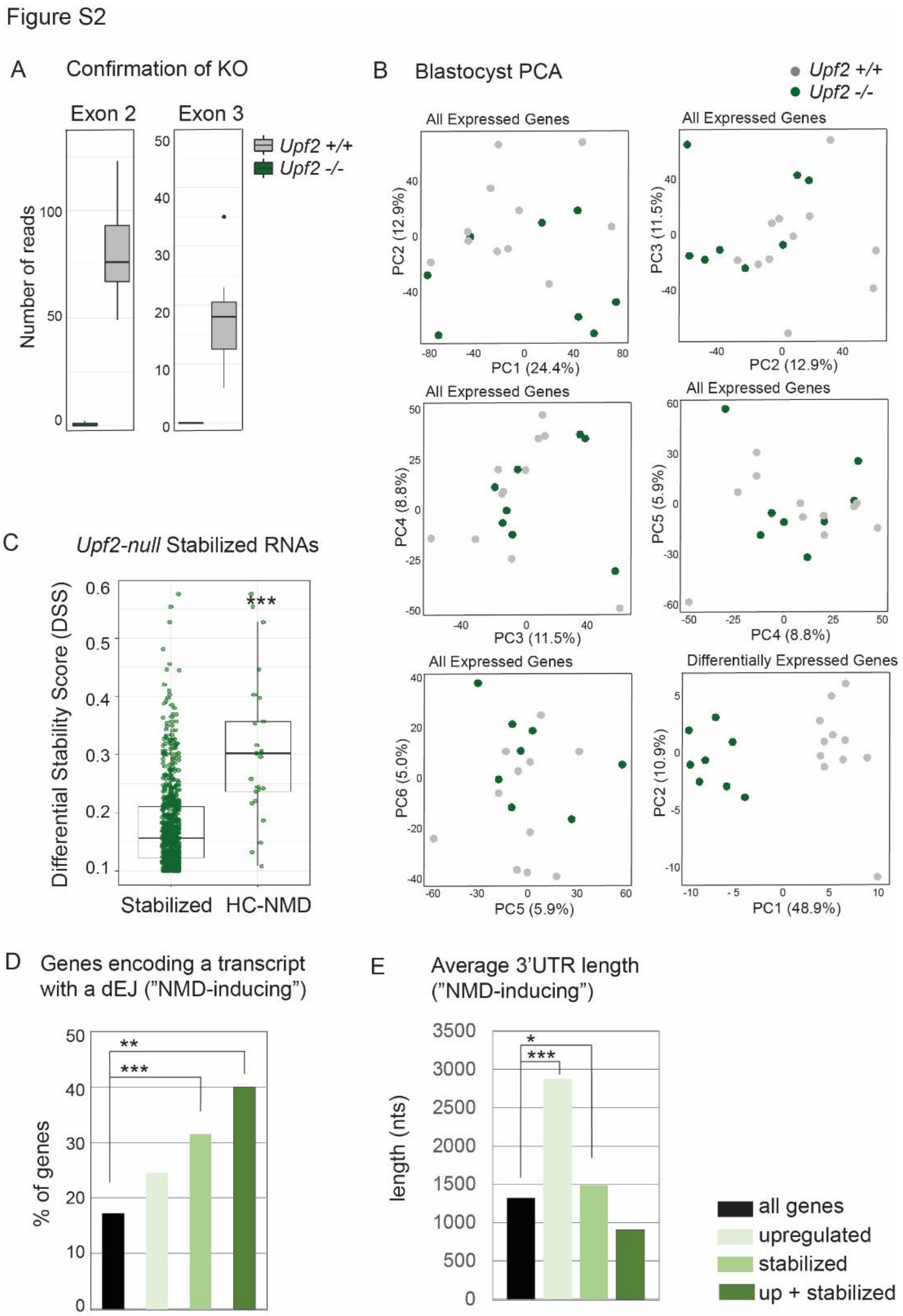

Fig. S2. Identification of NMD-regulated mRNAs in the mouse blastocyst. (A) Box plot of reads mapping to exon 2 and 3, deleted in *Upf2*-null embryos. **(B)** Principal component analysis demonstrates that *Upf2*-null and control blastocyst samples do not cluster separately in PCs 1-6 using all expressed genes. **(C)** Dot plot of mRNAs stabilized in *Upf2*-null blastocysts compared to controls, plotted by differential stability score (DSS) (Alkallas et al., 2017). Additionally plotted is the DSS for all high-confidence NMD targets (enriched for high DSS) (P=2.2e-05, Student's T-test). **(D)** The percent of total genes that encode a transcript with a dEJ ("NMD-inducing feature") is presented for all genes, genes upregulated in *Upf2*-null blastocysts, genes stabilized in *Upf2*-null blastocysts, and genes both upregulated and stabilized. Chi-squared analysis of proportions, ***P<0.001, **P<0.01. **(E)** As in (D), the average 3'UTR length ("NMD-inducing feature") is presented. Student's T-test, ***P<0.001, *P<0.05

Development • Supplementary information

Figure S3

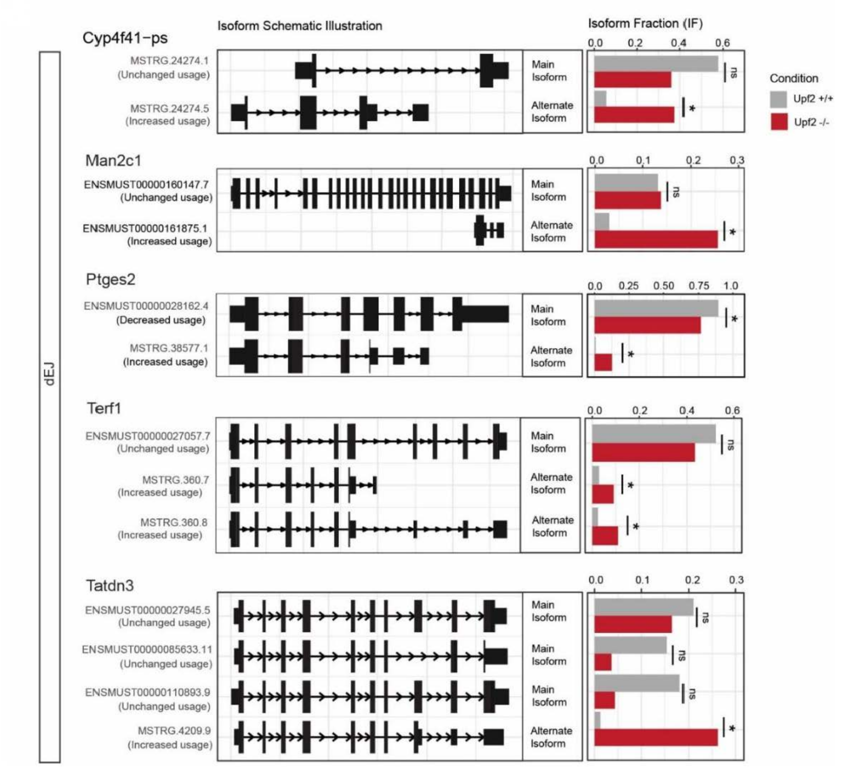

Fig. S3. Additional alternatively processed NMD mRNAs identified during mouse development. Isoform switch plots of selected genes with a dEJ (left), indicating the predominant isoform(s) and the alternate isoform expression in *Upf2*-null (red) and control (grey) blastocysts. Bar graphs on right indicate the isoform fraction. Adjusted p-value (Q): *** Q<0.001, ** Q<0.01; * Q<0.05; NS, not statistically significant.

Development • Supplementary information

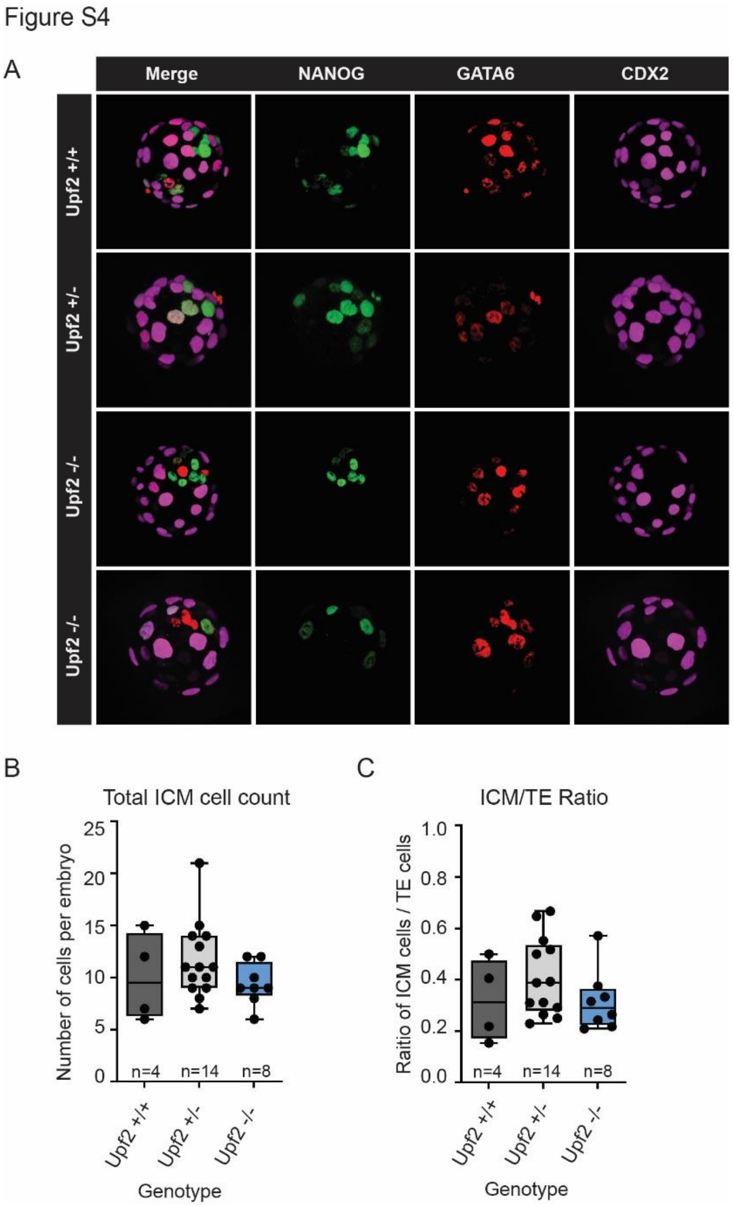

Fig. S4. Loss of Upf2 decreases total number of EPI cells in the blastocyst. (A) Additional 3D volume projections of *Upf2*-null and littermate control blastocysts stained for NANOG (EPI), GATA6 (PrE), and CDX2 (TE). **(B)** Box plot demonstrating the total number of ICM (NANOG- and GATA6-positive) cells per embryo (*Upf2*$^{+/+}$ vs. *Upf2*$^{-/-}$, P=0.36). **(C)** Box plot of the ICM/TE ratio for each genotype (*Upf2*$^{+/+}$ vs. *Upf2*$^{-/-}$, P=0.48). n=24 total blastocysts (n=8 *Upf2*$^{-/-}$, n=14 *Upf2*$^{+/-}$, n=4 Upf2$^{+/+}$).

Development • Supplementary information

Figure S5

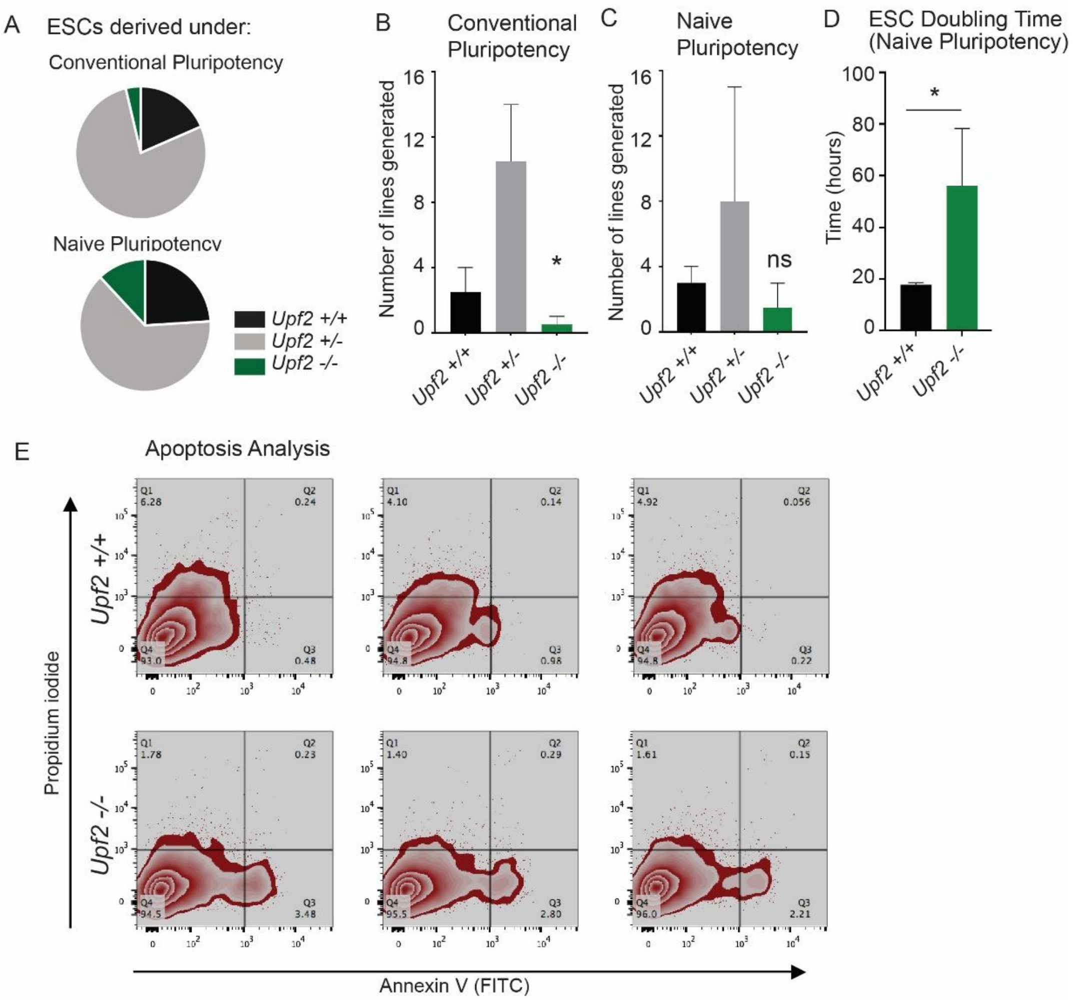

Fig. S5. Loss of Upf2 is incompatible with stable mESC derivation. (A) The proportion of ESC lines generated for each genotype under conventional pluripotency derivation conditions (serum-containing media with LIF) (Bryja et al., 2006) and naïve pluripotency conditions (2-inhibitor (2i, MEK inhibitor and GSK3 inhibitor) with LIF) (Czechanski et al., 2014). Under conventional and naïve conditions, 4% and 12% of lines generated were *Upf2-null,* respectively. **(B-C)** The total number of ESC lines (mean +/- SEM) derived under **(B)** conventional and **(C)** naïve conditions. *P<0.05, Student's T-test. **(D)** The time in hours (mean +/- SEM) required for ESCs (under naïve culture conditions) to double in number. *Upf2*-null ESCs proliferate significantly slower (n=3 *Upf2*-null, n=4 wild-type control). *P<0.05, Student's T-test. **(E)** Staining of propidium iodide and annexin V revealed a defect in apoptosis in *Upf2*-null lines maintained in naïve culture conditions, with an increase in the proportion of cells in Q3 (2.83% for *Upf2*-null vs. 0.56% for wild-type lines, n=3). P=0.02, Student's T-test.

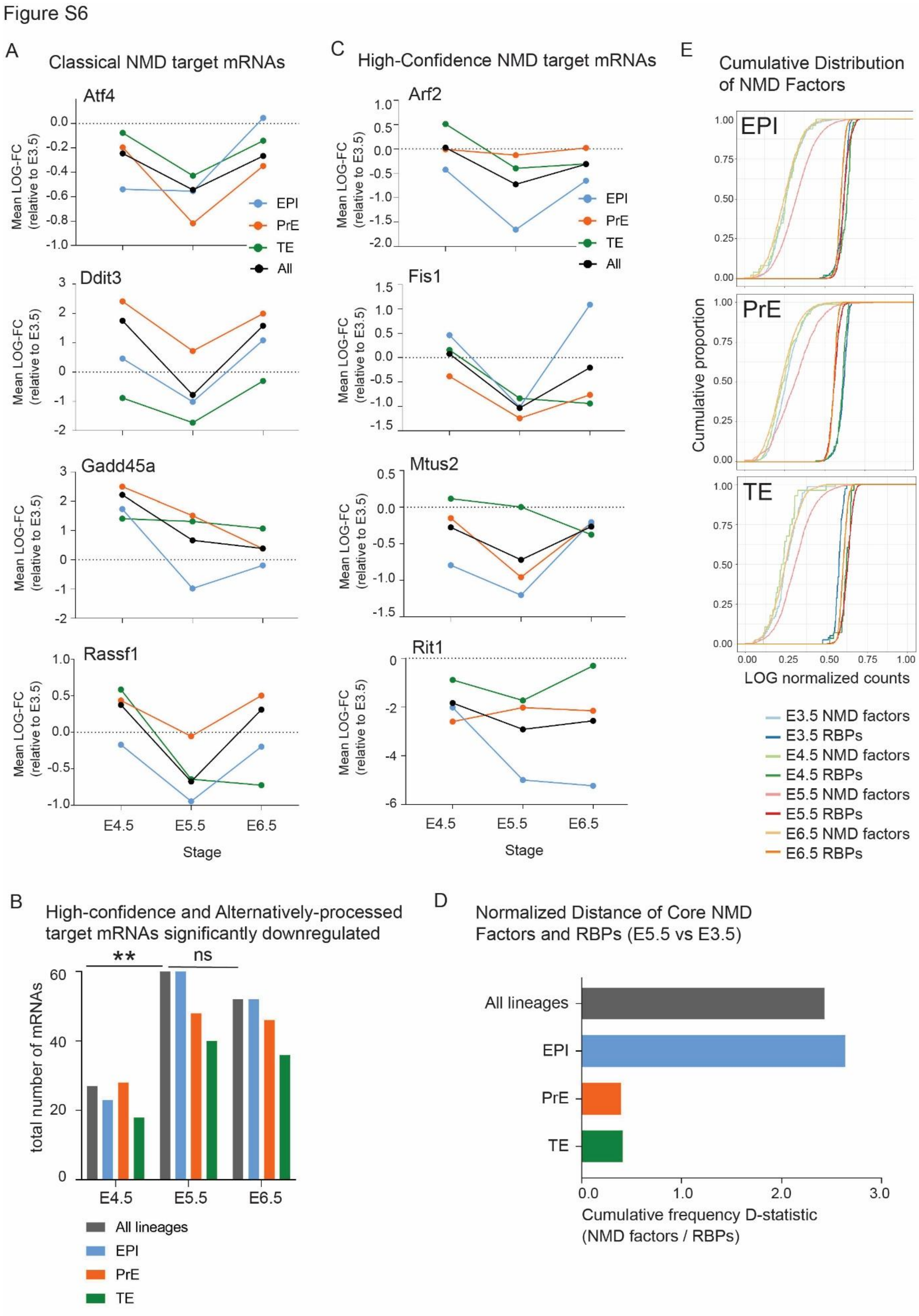

Development • Supplementary information

Fig. S6. NMD target mRNA and NMD factor expression reveals an upregulation of NMD magnitude at E5.5. (A) The expression of classical NMD target mRNAs is plotted from a scRNAseq dataset of mouse embryo cells (Nowotschin et al., 2019). Targets demonstrate a coordinated downregulation at E5.5, with the strongest regulation evident in the EPI, compared to other extra-embryonic lineages. **(B)** More than 70% of high-confidence and alternatively-processed NMD target mRNAs significantly decreased in expression, with the largest proportion downregulated between E4.5 (52%) and E5.5 (76%), and continued repression at E6.5 (70%). Chi-squared analysis of proportions, **P = 0.01, E5.5 vs E4.5, P = 0.43, E5.5 vs E6.5. **(C)** As in (A), the expression of representative NMD target mRNAs is presented, for targets defined as high-confidence in this study. **(D)** Plotted is a normalized measure of the shift in NMD factor expression between E3.5 and E5.5. Cumulative distribution frequencies were determined as in Fig 7E. The distance between curves (D) was calculated and normalized to the RBP distance. (see Methods). **(E)** Cumulative distribution plot of the normalized expression of NMD factors (n=11), as well as a set of RBPs (n=1066), at each stage of peri-implantation development. A large deviation in expression of NMD factors can be visualized at E5.5, but not at other stages.

References

Alkallas, R., Fish, L., Goodarzi, H., and Najafabadi, H.S. (2017). Inference of RNA decay rate from transcriptional profiling highlights the regulatory programs of Alzheimer's disease. Nat. Commun. *8*, 909.

Bryja, V., Bonilla, S., and Arenas, E. (2006). Derivation of mouse embryonic stem cells.

Czechanski, A., Byers, C., Greenstein, I., Schrode, N., Donahue, L.R., Hadjantonakis, A.-K., and Reinholdt, L.G. (2014). Derivation and characterization of mouse embryonic stem cells from permissive and nonpermissive strains. Nat. Protoc. *9*, 559–574.

Nowotschin, S., Setty, M., Kuo, Y.Y., Liu, V., Garg, V., Sharma, R., Simon, C.S., Saiz, N., Gardner, R., Boutet, S.C., et al. (2019). The emergent landscape of the mouse gut endoderm at single-cell resolution. Nature *569*.

Development • Supplementary information
