# Supplementary material for: Progression of the pluripotent epiblast depends upon the NMD factor UPF2
Source: Development. 2022 Nov 7;149(21):dev200764. doi: 10.1242/dev.200764 (PMC9687065; doi:10.1242/dev.200764)
Supplement: Supplementary file 1 [file develop-149-200764_review_history.pdf]

Progression of the pluripotent epiblast depends upon the NMD factor UPF2

Jennifer N. Chousal, Abhishek Sohni, Kristoffer Vitting-Seerup, Kyucheol Cho, Matthew Kim, Kun Tan, Bo Porse, Miles F. Wilkinson and Heidi Cook-Andersen
DOI: 10.1242/dev.200764

Editor: Maria Elena Torres-Padilla

Review timeline
Original submission: 23 March 2022
Editorial decision: 22 April 2022
First revision received: 28 July 2022
Editorial decision: 26 August 2022
Second revision received: 8 September 2022
Accepted: 9 September 2022

Original submission

First decision letter

MS ID#: DEVELOP/2022/200764

MS TITLE: Progression of the Pluripotent Epiblast Depends Upon the NMD Factor UPF2

AUTHORS: Jennifer N Dumdie, Abhishek Sohni, Kristoffer Vitting-Seerup, Kyucheol Cho, Matthew Kim, Kun Tan, Bo Porse, Miles F Wilkinson, and Heidi Cook-Andersen

Apologies for the time it has taken to get back to you with a decision on your above manuscript, but it has been difficult to find reviewers that take the assignment.

I have now finally received all the referees reports on the manuscript. The referees' comments are appended below, or you can access them online: please go to BenchPress and click on the 'Manuscripts with Decisions' queue in the Author Area.

The overall evaluation is positive and we would like to be able to publish a revised manuscript in Development, provided that the referees' comments can be addressed in full. Please attend to all of the reviewers' comments in your revised manuscript and detail them in your point-by-point response. If you do not agree with any of their criticisms or suggestions explain clearly why this is so. If it would be helpful, you are welcome to contact us to discuss your revision in greater detail. Please send us a point-by-point response indicating your plans for addressing the referee's comments, and we will look over this and provide further guidance.

Reviewer 1

Advance summary and potential significance to field

In this study Dumdie and colleagues investigate the role of the core NMD factor UPF2 in early mouse development.

© 2022. Published by The Company of Biologists under the terms of the Creative Commons Attribution License (https://creativecommons.org/licenses/by/4.0/).

First, the authors developed an early embryo Upf2 conditional knock-out mouse model. They observed that UPF2 is not required for developmental progression of the preimplantation embryo to form the blastocyst, but rather seems to act during peri-implantation development.

The authors went on to analyse transcripts that were upregulated and/or stabilised in Upf2-null blastocyst. This revealed that UPF2 regulates genes involved in cell cycle arrest and apoptosis, including the well characterised, Gadd45a. This is the first identification of NMD targets in the mouse blastocyst. In vitro growth assay shows that loss of Upf2 prevents cell proliferation and progression leading to cell death, thus being incompatible with ICM survival. The authors concluded that depletion of UPF2 leads to a deficit in epiblast cells and peri-implantation embryonic lethality. The authors went on to show that there is a transient shift in NMD activity during peri-implantation development and this correlates well with a change in the expression of number of canonical NMD factors.

In summary, this study provides a thorough characterisation of the role of the canonical NMD factor, UPF2, in the early embryo, precisely defining its role in the developmental progression of the ICM.

This study has been carefully planned and executed. The data is of high quality and supports the conclusions.

Comments for the author

Specific comments
- Is there any overlap between those RNAs shown to be regulated by UPF2 in Fig 2A and 3, with RNAs that have been shown to be regulated by UPF1? I am not aware of literature studying UPF1 regulation in the early embryo, but even if this has not been done, what is the overlap with RNA-seq and/or CLIP data of UPF1 later in development?
- How many of the phenotypes observed are predicted to be specific for Upf2, or are rather common to all core NMD factors?
- The authors should discuss and/or speculate on the mechanisms that drive an increase in the expression of NMD factors at E5.5.
- In the well-written and comprehensive Introduction, when referring to loss of NMD factors causing early embryonic lethality, while this applies for instance to mice and zebrafish, the author may want to distinguish this from C. elegans. It has been shown by many labs that mutations in core NMD factors (Smg 1-7) give rise to vulval phenotypes but not embryonic lethality. There are, however, other nematode NMD factors, such as Smgl-1, 2 (mammalian homologs NBAS and DHX324),
which are essential for viability in nematodes.
- When describing NMD targets (line 190) the authors may wish to include a recent study that provides RNA-seq analysis of HeLa cells upon UPF1 knock-down and compared to NBAS depletion (Longman et al. (2020) Genes Dev)

Reviewer 2

Advance summary and potential significance to field

Dumdie et al. investigate the role of NMD in early mammalian development.

NMD has been recognized previously as an evolutionary conserved pathway that degrades several types of mRNAs in animal cells. While it has been shown to be essential for early development of several organisms, including mice, the details are unknown. The authors use a previously generated and validated Upf2 KO mouse model throughout the study. The authors show that Upf2-/- embryos fail at peri-implantation stages. The authors then use transcriptomics of blastocysts combined with rigorous bioinformatic analyses to define a list of NMD targets in early mouse embryos, which suggest that NMD supports cell survival and downregulates apoptotic pathways. Further analyses of early mouse embryos revealed that NMD is specifically required for Epi survival, as opposed to PrE or TE in blastocysts.

© 2022. Published by The Company of Biologists under the terms of the Creative Commons Attribution License (https://creativecommons.org/licenses/by/4.0/).

The authors use existing single-cell RNA-seq datasets to show that NMD factors peak in expression at E5.5 and their corresponding targets are lowest at this time-point in an Epi-specific manner.

Overall, this study is well executed and sheds novel insights into the role of NMD in early mammalian development. The following critical points should however be addressed:

Comments for the author

Major points to be addressed:

- Line 162: PCA analysis will cluster samples based on shared features/changes in gene expression, even if there are no global changes in gene expression (as in increased or decreased total mRNA). The observed lack of clustering in the principal components shown here suggests that there may not be shared changes in gene expression across all KO vs all WT samples. That's being said, the shown principal components explain only ~40% of the difference suggesting the presence of perhaps more dominants PCs. Please report other PCs.
- Line 355: the argument that Epi cells fail to survive could be greatly supported by staining for phospho-histoneH3 as a proliferation marker, and caspase3 as an apoptosis marker followed by quantification to see whether cells do not divide or die. Additionally, collecting and staining blastocysts at E4.5 for lineage markers may reveal a more severe loss of Epi at E4.5 prior to implantation. These are relatively straightforward experiments that may be very important to the story.

Minor points:

- Line 108: “to revealed” typo.
- Line 115: While this mutant seems to produce a loss of function, it leaves a truncated protein. It may be best to mention that here.
- Line 130, Figure 1 C-D: arrange panels in chronological manner.
- Line 133: “This suggests …” rewrite sentence for clarity.
- Line 151: Please highlight that this mutant cannot rule out a maternal contribution of Upf2 to early pre-implantation stages. If it’s possible to immunostain blastocysts for it, please do so to rule that out.
- Line 163: “.. did not result in a global shift in gene expression”. This claim cannot be made without a spike-in corrected transcriptomic analysis, which is not the case here. Please remove.
- Line 355: Figure 5B: Show numbers of embryos used in figures in addition to legends. It would be great if single data points are shown on the plot for transparent reporting.
- Line 390: “22 NMD factors which expression is assessed”. Please add a table with the factors used if not added to supplements already.
- Line 390: Is it possible to validate one or two of these factors via immunostaining in E3.5 vs E4.5/E5.5 wild type embryos to show increased expression in situ?

Reviewer 3

Advance summary and potential significance to field

In this set of well-designed experiments, the authors demonstrate that nonsense-mediated RNA decay (NMD) has stage- and lineage-specific developmental functions in the early mammalian embryo. Previous studies established that loss or depletion of NMD causes early embryo lethality in diverse organisms. However, in mice, the timing and underlying molecular mechanisms of lethality remain unknown. Like in my other biological processes, proteome regulation in early embryonic brain development has been mostly studied at the level of transcription, so mRNA stability has not been demonstrated. In this manuscript to determine the role of NMD pathway in early mouse embryo development, the authors targeted UPF2, one of the major factors of NMD machinery. They found that UPF2 is dispensable at pre-implantation stage to form the blastocyst but essential peri-implantation. They then performed an RNAseq at this stage and found that genes downregulated upon knockout of Upf2 regulates embryo development and pluripotency. Genes upregulated in <I>*Upf2*</I>-null blastocysts are enriched for apoptosis and cell cycle arrest. Most importantly, the authors identified high-confidence NMD target mRNAs in inner cell mass and epiblast in the blastocyst stage.

© 2022. Published by The Company of Biologists under the terms of the Creative Commons Attribution License (https://creativecommons.org/licenses/by/4.0/).

Comments for the author

I only have minor comments:
1) The last sentence of introduction (…to revealed…) may need correction
2) Describe the PCA abbreviation when first mentioned
3) In the discussion, can authors also speculate if disruption of other essential NMD factors would cause similar phenotypes and gene expression changes
4) Be consistent with the italic use of <I>*Upf2*</I>-null throughout the text (i.e., Figure 1 legend, first sentence)

First revision

Author response to reviewers' comments

Reviewer 1:

In this study Dumdie and colleagues investigate the role of the core NMD factor UPF2 in early mouse development. First, the authors developed an early embryo Upf2 conditional knock-out mouse model. They observed that UPF2 is not required for developmental progression of the preimplantation embryo to form the blastocyst, but rather seems to act during peri-implantation development.

The authors went on to analyse transcripts that were upregulated and/or stabilised in Upf2-null blastocyst. This revealed that UPF2 regulates genes involved in cell cycle arrest and apoptosis, including the well characterised, Gadd45a. This is the first identification of NMD targets in the mouse blastocyst. In vitro growth assay shows that loss of Upf2 prevents cell proliferation and progression leading to cell death, thus being incompatible with ICM survival. The authors concluded that depletion of UPF2 leads to a deficit in epiblast cells and peri-implantation embryonic lethality. The authors went on to show that there is a transient shift in NMD activity during peri-implantation development and this correlates well with a change in the expression of number of canonical NMD factors. In summary, this study provides a thorough characterisation of the role of the canonical NMD factor, UPF2, in the early embryo, precisely defining its role in the developmental progression of the ICM. This study has been carefully planned and executed. The data is of high quality and supports the conclusions.

Specific comments

Is there any overlap between those RNAs shown to be regulated by UPF2 in Fig 2A and 3, with RNAs that have been shown to be regulated by UPF1?
Response: We found some overlap between the blastocyst high-confidence NMD target mRNAs that we identified and mouse NMD target mRNAs identified by other studies. These studies identified NMD targets in a variety of ways, not only via perturbation of UPF1 (Hurt et al., *Genome Res* 2013), but also other NMD factors, including SMG1 (McIlwain et al., *Proc Natl Acad Sci* 2010) and RBM8 (Mao et al., *RNA* 2017). The blastocyst NMD targets that overlap with targets identified by other studies are shown in **Figure 3A-B** of the revised manuscript. More details of our overlap analysis (including the specific studies previously identifying mouse NMD targets) are in **Supplemental Table S2** (See "Overlap" tab). The relevant section of the text is copied below:

> Lines 201-211: "*Our final approach to identify bona fide NMD target mRNAs in blastocysts was to cross-reference with mouse mRNAs previously implicated as NMD targets. To achieve this, we established a list of putative and high-confidence mouse NMD target mRNAs defined in various different biological contexts; e.g., cell lines and tissues (Bao et al., 2015; Hurt et al., 2013; Mao et al., 2015; McIlwain et al., 2010; Mooney et al., 2017; Thoren et al., 2010; Weischenfeldt et al., 2012). Comparison with this list revealed a 35% overlap with genes upregulated in Upf2-null blastocysts, and 30% overlap with NMD- destabilized mRNAs (Table S2). This incomplete overlap is expected and does not negate the validity of the other putative NMD targets identified, as NMD target mRNAs are known to vary across cell types, in different*

© 2022. Published by The Company of Biologists under the terms of the Creative Commons Attribution License (https://creativecommons.org/licenses/by/4.0/).

biological contexts, and to respond to different NMD branch-specific factors (Fatscher et al., 2015; Huang and Wilkinson, 2012; Huang et al., 2011; Zetoune et al., 2008). "

I am not aware of literature studying UPF1 regulation in the early embryo, but even if this has not been done, what is the overlap with RNA-seq and/or CLIP data of UPF1 later in development?
Response: To our knowledge, no studies have been conducted to identify UPF1-bound or UPF1-regulated mRNAs at any stage of mouse development.

How many of the phenotypes observed are predicted to be specific for Upf2, or are rather common to all core NMD factors?
Response: Knockout of many different NMD factors (UPF1, UPF2, UPF3A, SMG1, and SMG6) in mice leads to early embryonic lethality during the peri-implantation period and possibly the post-implantation period. Thus, this is a common phenotype. However, it is difficult to make any conclusions beyond that, as previous studies that have knocked out NMD factor genes have not determined the precise timing of embryonic lethality, nor have they deciphered specific developmental steps affected by NMD factor knock-out. We explicitly state this and discuss the possibility of a unifying theme in the revised Discussion:

> Lines 435-450: *"A key question is whether our results with mice lacking UPF2 are generalizable for other NMD factors. While previous studies that have knocked out NMD factor genes during mouse development have not determined the precise timing of embryo lethality, they point towards a common defect in the peri-implantation period (Hwang and Maquat, 2011; Li et al., 2015; Mao et al., 2015; McIlwain et al., 2010; Medghalchi et al., 2001; Shum et al., 2016; Weischenfeldt et al., 2008). In the case of SMG1, its loss results in smaller embryos with death by early post-implantation development (McIlwain et al., 2010). Similar to our phenotypic data, Upf1-null (Medghalchi et al., 2001) and Smg6-null blastocysts (Li et al., 2015) undergo regression after a short period of culture. Also similar, we have shown that Upf3a-null blastocysts display developmental defects with death in the peri-implantation period (Shum et al., 2016). Here, we traced the role of NMD in the peri-implantation window to the EPI specifically and uncovered NMD target mRNAs and a stage-specific shift in NMD activity. An enticing hypothesis that unifies these findings is that the NMD pathway serves as a regulatory pathway within the EPI around the time of implantation, allowing for shifts in cohorts of mRNAs critical for this pluripotent cell type. In the future, it will be important to test this hypothesis by conducting rigorous analysis of mice lacking different NMD factors. "*

The authors should discuss and/or speculate on the mechanisms that drive an increase in the expression of NMD factors at E5.5.
Response: We have added the following comment to the Discussion:

> Lines 534-542: "*While we do not know the mechanism responsible for this transient increase in NMD magnitude, we found that a large cohort of NMD factors are transiently upregulated at the same embryonic time point, consistent with their upregulation being responsible for the increased NMD magnitude (Figures 6 and S6). The mechanism responsible for this coordinated upregulation of many NMD factors is an interesting future question; one possibility is that these NMD factor genes are all upregulated by a common transcription factor responsive to a stage-specific developmental cue. Future studies are necessary to tease apart how coordinated regulation on this scale is mediated to control development."*

In the well-written and comprehensive Introduction, when referring to loss of NMD factors causing early embryonic lethality, while this applies for instance to mice and zebrafish, the author may want to distinguish this from C. elegans. It has been shown by many labs that mutations in core NMD factors (Smg 1-7) give rise to vulval phenotypes but not embryonic lethality. There are, however, other nematode NMD factors, such as Smgl-1, 2 (mammalian homologs NBAS and DHX324), which are essential for viability in nematodes.
Response: We agree that a more comprehensive review of the literature on this topic is important. Thus, in the revised manuscript, we have added a paragraph on the possible conserved and non-conserved roles of NMD in early development:

© 2022. Published by The Company of Biologists under the terms of the Creative Commons Attribution License (https://creativecommons.org/licenses/by/4.0/).

Lines 443-468: *"Another interesting future question is whether NMD has conserved roles in early embryo development that extend beyond mice. In zebrafish, knockdown of any of a number of different NMD factors causes severe developmental defects, including perturbations in early patterning, as well as reduced viability (Anastasaki et al., 2011; Wittkopp et al., 2009). It is likely that human embryonic development also depends on NMD, as there have been no reports of homozygous mutations in core NMD genes in humans, with the exception of UPF3B, which is not essential for NMD (Jaffrey and Wilkinson, 2018). In Drosophila, Upf1 and Upf2 are required for larval viability, in part because NMD confers a competitive growth advantage to fly embryonic cells (Metzstein and Krasnow, 2006). Given that NMD is known to drive the proliferation of several mammalian cell types (Azzalin and Lingner, 2006; Gehen et al., 2008; Lou et al., 2014; Lykke-Andersen and Jensen, 2015; Weischenfeldt et al., 2008), it is tempting to speculate that a conserved function of NMD is to drive the expansion of specific cell populations during early development. The role of NMD in C. elegans appears to be more complex, as knockdown of the NMD genes, Nbas and Dhx34, causes severe developmental defects (Longman et al., 2007), whereas complete knockout of several other NMD genes, including core NMD genes, only cause male bursa and hermaphrodite vulva defects in C. elegans (Pulak and Anderson, 1993)."*

When describing NMD targets (line 190) the authors may wish to include a recent study that provides RNA-seq analysis of HeLa cells upon UPF1 knock-down and compared to NBAS depletion (Longman et al. (2020) Genes Dev) .
Response: Many important studies, including Longman et al., have identified putative NMD target mRNAs in *human* cells. However, since our study identified mouse NMD targets, and most NMD target mRNAs are not conserved (even between mammalian species), we focused our analyses only on previously identified *mouse* NMD target mRNAs (see our comments on this topic above). We modestly revised the first sentence of this paragraph to make this more clear:

Lines 201-202: *"Our final approach to identify bona fide NMD target mRNAs in blastocysts was to cross-reference with mouse mRNAs previously implicated as NMD targets."*

Reviewer 2:

Dumdie et al. investigate the role of NMD in early mammalian development. NMD has been recognized previously as an evolutionary conserved pathway that degrades several types of mRNAs in animal cells. While it has been shown to be essential for early development of several organisms, including mice, the details are unknown. The authors use a previously generated and validated Upf2 KO mouse model throughout the study. The authors show that Upf2-/- embryos fail at peri-implantation stages. The authors then use transcriptomics of blastocysts combined with rigorous bioinformatic analyses to define a list of NMD targets in early mouse embryos, which suggest that NMD supports cell survival and downregulates apoptotic pathways. Further analyses of early mouse embryos revealed that NMD is specifically required for Epi survival, as opposed to PrE or TE in blastocysts. The authors use existing single-cell RNA-seq datasets to show that NMD factors peak in expression at E5.5 and their corresponding targets are lowest at this time- point in an Epi-specific manner. Overall, this study is well executed and sheds novel insights into the role of NMD in early mammalian development. The following critical points should however be addressed:

Major points to be addressed:

Line 162: PCA analysis will cluster samples based on shared features/changes in gene expression, even if there are no global changes in gene expression (as in increased or decreased total mRNA). The observed lack of clustering in the principal components shown here suggests that there may not be shared changes in gene expression across all KO vs all WT samples. That's being said, the shown principal components explain only ~40% of the difference, suggesting the presence of perhaps more dominants PCs. Please report other PCs.
Response: We performed analysis of PCs 1-6 using all expressed genes within our dataset, and found that this accounts for ~70% of the variance. We did not observe clustering of our knock-out and wild-type samples using these components. However, when we instead did clustering analysis using the top-100 differentially expressed genes, we observed good separation between groups. See new **Figure S2B**, which is copied below:

© 2022. Published by The Company of Biologists under the terms of the Creative Commons Attribution License (https://creativecommons.org/licenses/by/4.0/).

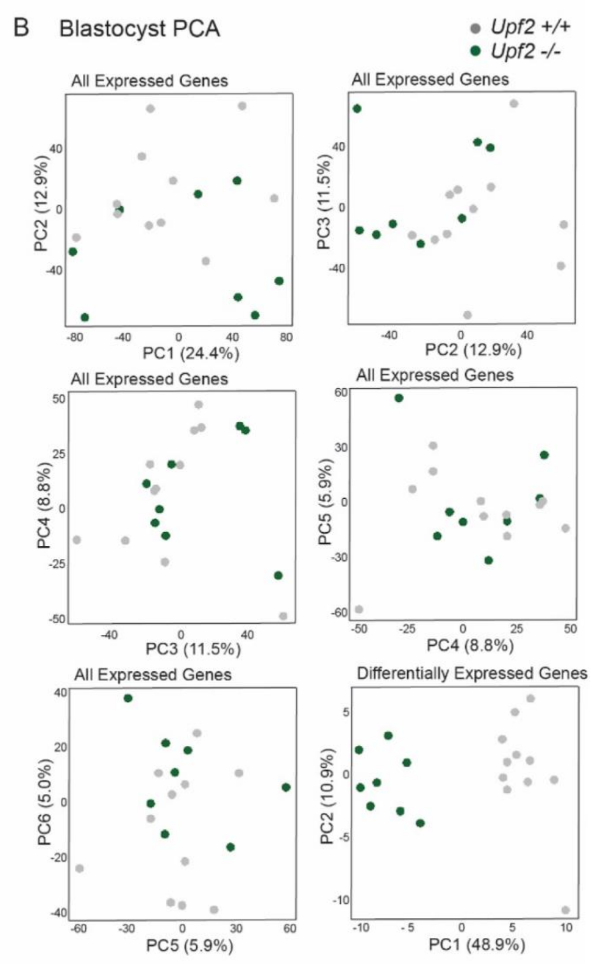

Line 355: the argument that Epi cells fail to survive could be greatly supported by staining for phospho-histoneH3 as a proliferation marker, and caspase3 as an apoptosis marker followed by quantification to see whether cells do not divide or die. Additionally, collecting and staining blastocysts at E4.5 for lineage markers may reveal a more severe loss of Epi at E4.5 prior to implantation. These are relatively straightforward experiments that may be very important to the story.

Response: We tried a number of approaches to assay the nature of the defect in EPI progression during implantation (E3.5 to E5.5). For example, to assay apoptosis, we performed TUNEL analysis and IF of active caspase 3 expression in NANOG+ cells in blastocysts before and during *in vitro* outgrowth. At E3.5, we observed far fewer NANOG+ cells in the KO (only ~5 EPI cells per embryo, as in **Figure 5**), and thus it was not feasible to identify enough active caspase 3+ or TUNEL+ EPI (NANOG)+ cells to draw conclusions. When we cultured these blastocysts for 24 to 48 hours to focus on slightly later stages when defects in the KO are more readily apparent, no NANOG+ positive cells were detectable in KO embryos, so we could not assay apoptosis.

As an alternative approach to address this question, we elected to use an *in vitro* system to allow us to assay both cell proliferation and apoptosis with a larger number of cells. As detailed in the revised manuscript, we were able to use naïve (but not primed) ESC-derivation conditions to establish *Upf2*-null ESC lines. *Upf2*-null lines displayed a significant defect in proliferation compared with littermate control (WT and heterozygous) lines), with a doubling time more than 2-fold that of controls. *Upf2*-null naïve ESCs also showed significantly increased rates of apoptosis, as assessed by Annexin V and FACS sorting. Given that pluripotency exists as a continuum from naïve (more representative of EPI in the late blastocyst) to primed (more representative of EPI at post-implantation stages), our results suggest a greater requirement for NMD at later stages of ESC maturation. This is consistent with our findings in the blastocyst. These new data are presented in the following text and new figures:

© 2022. Published by The Company of Biologists under the terms of the Creative Commons Attribution License (https://creativecommons.org/licenses/by/4.0/).

Lines 304-326 and new Figure S5A-E: *"To further assess this EPI defect, we asked whether Upf2-null blastocysts can generate embryonic stem cells (ESCs). In support of an EPI defect, we were not able to derive stable Upf2-null ESC lines using conventional derivation conditions with serum and LIF-containing media (Bryja et al., 2006). Thus, despite Upf2-null blastocysts being present at the initiation of culture at a normal Mendelian ratio (Figure S1D), only one Upf2-null blastocyst initiated growth (of 28 lines from all genotypes), and it died with the first passage (Figure S5A,B). In contrast, ESC lines were generated from littermate control blastocysts at a normal Mendelian ratio (Figure S5A,B).*

To probe the timing of the EPI defect in the Upf2-null blastocyst, we assessed whether these blastocysts could instead give rise to ESCs at a more naïve stage. It is well- established that naïve (or "ground state") ESCs derived under 2-inhibitor (2i) (MEK inhibitor + GSK3 inhibitor) conditions (Czechanski et al., 2014) most closely resemble the early, unrestricted pre-implantation EPI at E3.75-4.5 (Boroviak and Nichols, 2014). In contrast, "primed ESCs," which are derived under conventional conditions (with serum and LIF) instead represent post-implantation EPI cells (E5.5-E6.5) that are about to enter gastrulation (Buecker et al., 2014; Hayashi et al., 2011). Using 2i-derivation conditions, we found that 12% (3 out of 25) of ESC lines generated from blastocysts obtained from Upf2-heterozygous matings were Upf2-null (Figure S5A-C). While 12% is less than the expected Mendelian ratio of 25%, our ability to derive several Upf2-null naïve ESCs indicates that UPF2 is not absolutely required for the naïve stage. Nonetheless, we found Upf2-null naïve ESCs were abnormal, exhibiting slower cell expansion and increased apoptosis relative to littermate control lines grown in parallel (Figures S5D,E). Together, these results—that UPF2 promotes the proliferation and survival of naïve ESCs and is critical for the progression and/or survival of ESCs cells to the primed state—support that UPF2 has stage-specific roles in EPI cells in the blastocyst."

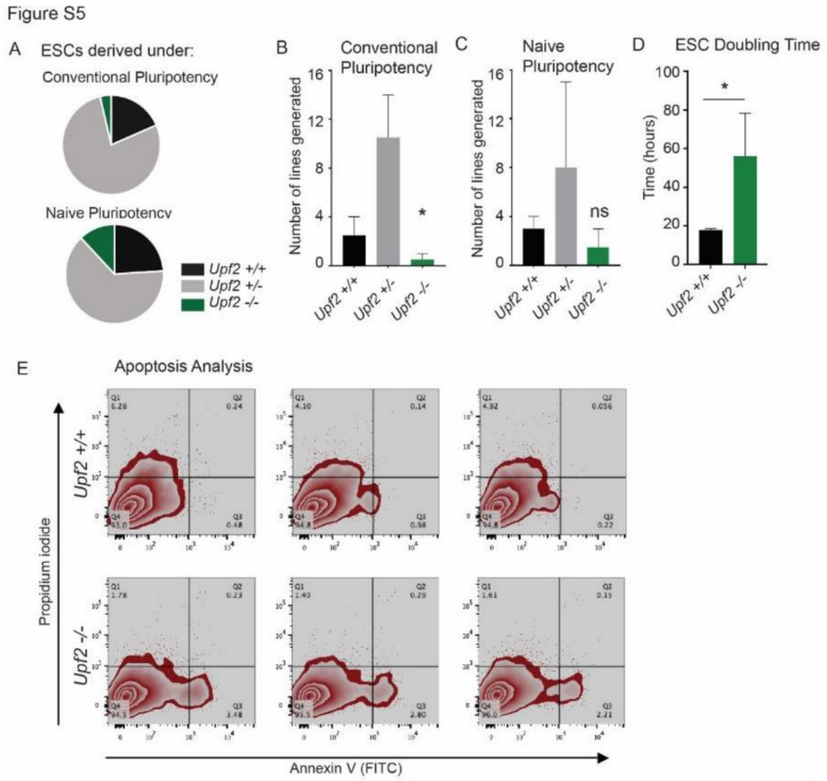

Figure S5: Loss of Upf2 is incompatible with stable mESC derivation. (A) The proportion of ESC lines generated for each genotype under conventional pluripotency derivation conditions (serum-containing media with LIF) (Bryja et al., 2006) and naïve pluripotency conditions (2- inhibitor (2i, MEK inhibitor and GSK3 inhibitor) with LIF) (Czechanski et al., 2014). Under conventional and naïve conditions, 4% and 12% of lines generated were *Upf2-null*, respectively. **(B-C)** The total number of ESC lines (mean +/- SEM) derived under **(B)** conventional and **(C)** naïve conditions. *P<0.05, Student's T-test. **(D)** The time in hours (mean +/- SEM) required for ESCs (under naïve culture conditions) to double in number. *Upf2*-null ESCs proliferate significantly slower (n=3 *Upf2*-null, n=4

© 2022. Published by The Company of Biologists under the terms of the Creative Commons Attribution License (https://creativecommons.org/licenses/by/4.0/).

wild-type control). *P<0.05, Student's T-test. **(E)** Staining of propidium iodide and annexin V revealed a defect in apoptosis in *Upf2*-null lines maintained in naïve culture conditions, with an increase in the proportion of cells in Q3 (2.83% for *Upf2*-null vs. 0.56% for wild-type lines, n=3). P=0.02, Student's T-test.
Minor points:

Line 108: "to revealed" typo.
Response: This has been corrected:

Line 115: While this mutant seems to produce a loss of function, it leaves a truncated protein. It may be best to mention that here.
Response: We have added the fact that a truncated UPF2 protein is produced with this mutant line here for clarity:

> Line 104-106: "*Recombination results in deletion of exons 2 and 3 (Figure S1B), which generates a truncated protein lacking NMD activity (Weischenfeldt et al., 2008)*".

Line 130, Figure 1 C-D: arrange panels in chronological manner.
Response: The reason that this embryonic lethality/phenotypic defect temporal data is discussed in reverse-chronological order is because this is the order in which we performed the experiments. We also think this order of presentation follows logically in the text. However, if the reviewer feels otherwise, we can revise these results so they are presented in a chronological order.

Line 133: "This suggests …" rewrite sentence for clarity.
Response: Thank you for pointing this out. This point has been edited for clarity:

> Previous version: "*The total implantation sites per female was within the normal range for wild-type C57/BL6 mice (Flores et al., 2014) and was not different between E5.5 and E6.5 (Figure 1B, S1C). This suggests there were not a significant number of Upf2-null embryos that initiated but ultimately failed to undergo implantation, not detected during our isolations*".
> **Revised version** Lines 117-122: "*Implantation sites and resorption sites per female from Upf2-heterozygous crosses were within the normal range observed for wild-type C57/BL6 mice (Flores et al., 2014) and not different between E5.5 and E6.5 (Figure 1B-D, S1C). Although it was not possible to genotype resorbed embryos because of the limited amount of embryonic DNA recovered, these findings suggest that Upf2-null embryos do not have an abnormally high resorption rate.*"

Line 151: Please highlight that this mutant cannot rule out a maternal contribution of Upf2 to early pre-implantation stages. If it's possible to immunostain blastocysts for it, please do so to rule that out.
Response: It is true that loss of the maternal contribution could lead to an even more severe phenotype than what we observed. Unfortunately, the only good UPF2 antibody that we are aware of was made against the C-terminal 206 amino acids (Lykke-Andersen et al. *Cell*, 2000), and thus would not be able to detect the difference between normal UPF2 and the truncated UPF2 produced by the *Upf2*-null mutant. Thus, it is not possible to immunostain blastocysts for maternal UPF2. We have added this consideration to the Discussion:

> Lines 450-452: ***"Of note, the phenotypic defects we observed in Upf2-null mice may be an underestimate of UPF2's early embryonic functions, as maternal UPF2 inherited from the oocyte could mask and/or reduce embryonic defects. "***

Line 163: ".. did not result in a global shift in gene expression". This claim cannot be made without a spike-in corrected transcriptomic analysis, which is not the case here. Please remove.
Response: We agree that a spike-in control is required to definitively know whether *Upf2* KO causes a global shift in mRNA level. We deem this unlikely because PCA using the full repertoire of expressed genes did not detect large differences in gene expression in the top principal components examined (see related comments in response to Reviewer 1). We have revised our comments about our PCA as follows:

> Lines 138-141: "*Consistent with no overt phenotypic defects in the early blastocyst, plotting the principal components that represent the most variance in our dataset did not lead to clustering of Upf2-/- (n=8) and Upf2+/+ (n=11) samples. Instead, we found that loss of UPF2 affected the expression of a specific cohort of mRNAs (Figure S2B).* "

© 2022. Published by The Company of Biologists under the terms of the Creative Commons Attribution License (https://creativecommons.org/licenses/by/4.0/).

Line 355: Figure 5B: Show numbers of embryos used in figures in addition to legends. It would be great if single data points are shown on the plot for transparent reporting.
Response: Thank you for this suggestion to improve clarity. All summary boxplots for immunofluorescence analysis have been amended to include individual embryo data points, with total embryo numbers specified both in the figure and in the figure legends (n=24 total blastocysts (n=8 *Upf2*$^{-/-}$, n=14 *Upf2*$^{+/-}$, n=4 Upf2$^{+/+}$).). See revised Figures 5, S4, copied below:

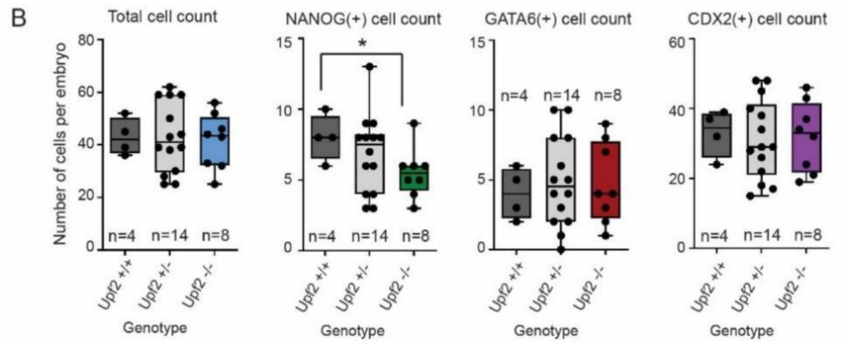

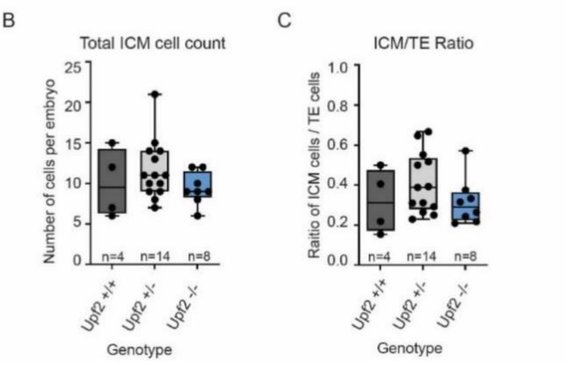

Line 390: "22 NMD factors which expression is assessed". Please add a table with the factors used if not added to supplements already.
Response: We apologize that we did not make it clear in the original submission that this list of NMD factors is in **Figure 6D**. We have now added this clarification to the text:

> Line 350-354: "*One mechanism by which NMD magnitude could be upregulated is through upregulation of NMD factors. To test this, we examined the temporal expression pattern of the 22 NMD factor genes listed in Figure 6D (Chan et al., 2009; Chang et al., 2007; Kishor et al., 2019; Kurosaki et al., 2019; Longman et al., 2013; Palma et al., 2021; Popp and Maquat, 2013; Schweingruber et al., 2013).*"

Line 390: Is it possible to validate one or two of these factors via immunostaining in E3.5 vs E4.5/E5.5 wild type embryos to show increased expression in situ?
Response: We feel it is not feasible to do this immunostaining experiment given the difficulty of isolating E4.5/E5.5 embryos and the number of embryos it would take to validate several NMD factors. Additionally, if we performed immunostaining analysis on only 1 or 2 NMD factors, this might not be convincing. Thus, we elected to instead address this question by leveraging a published ESC proteomic dataset obtained from ESCs cultured across a window of differentiation comparable with the implantation period *in vivo* (E3.5-E6.5). This validated the coordinated change in expression of all NMD targets (as a group) at the protein level. This approach also validated changes in NMD factor expression at the protein level. Together, these data verify the transient shift in NMD magnitude we observe during this critical time in embryo development. The relevant text and new figures are copied below:

> Lines 371-386 and new Figure 6 F-G: "*To independently assess this shift in NMD magnitude, we turned to an ESC proteomics dataset generated by Yang et al. (Yang et al., 2019). The time points analyzed model EPI differentiation from the blastocyst to peri- implantation stages, the same temporal period exhibiting defects in Upf2-null embryos. In particular, the*

© 2022. Published by The Company of Biologists under the terms of the Creative Commons Attribution License (https://creativecommons.org/licenses/by/4.0/).

ESCs used by this study are naïve ESCs (which resemble pre-implantation (E3.75-4.5) EPI cells) (Boroviak and Nichols, 2014) that were cultured to form epiblast- like cells (EpiLCs) (which most closely resemble post-implantation pre-gastrulation (E5.5- E6.5) EPI cells) (Buecker et al., 2014; Hayashi et al., 2011). We examined the expression of proteins encoded by the high-confidence blastocyst NMD target RNAs we identified (Figure 3) and found that their temporal expression mirrored their expression at the RNA level. Specifically, these proteins exhibited significantly reduced expression during the progression from naïve ESCs to EpiLCs (Figure 6F, top). NMD factors, as a group, exhibited an inverse pattern, as predicted (Figure 6F, bottom). The core NMD factors, UPF1 and UPF2, exhibited a particularly robust increase in expression, with significant increases at in vitro time points that corresponded to ~E5.5 in vivo (36 and 48 hours) (Figure 6F, bottom). We conclude that NMD magnitude is increased in the early embryo at E5.5, leading to destabilization of many NMD target mRNAs and a corresponding decrease in the expression of their encoded proteins."

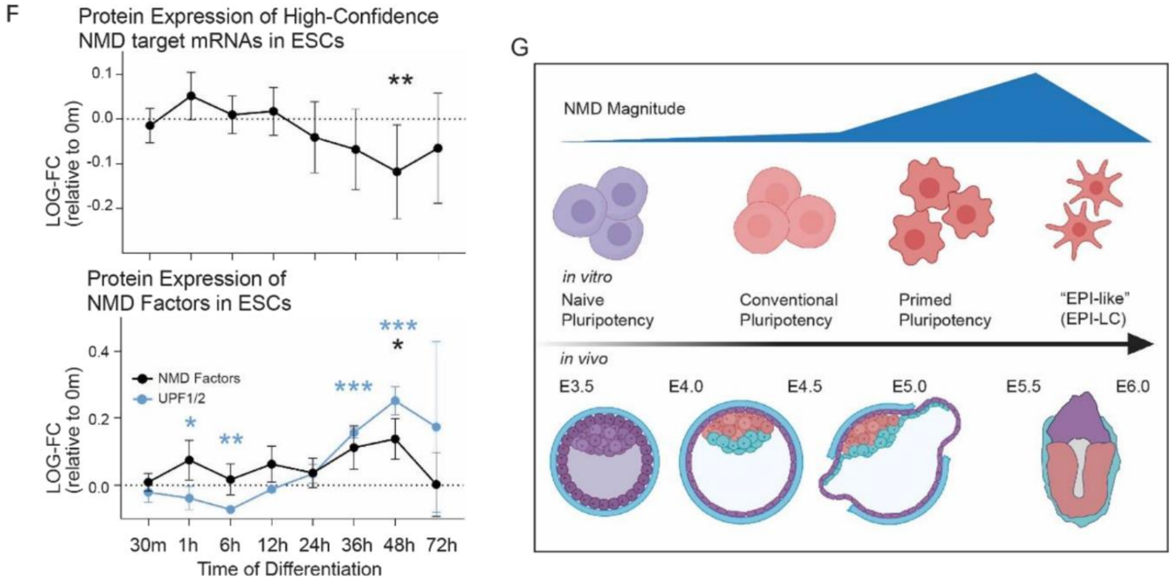

Figure 6 Legend: (F) Using a published mESC proteomics dataset, we evaluated the expression of NMD targets and factors across the window of differentiation from naïve (0m) to EpiLCs (~72h) (Yang et al., 2019). The log(fold change) of proteins encoded by high-confidence and alternatively processed NMD targets (n=75) (top) and core NMD factors (n=14) (bottom) is plotted. NMD targets significantly decrease and NMD factors significantly increase after 48h of differentiation, relative to 0m. (*P<0.05, **P<0.01, ***P<0.001). **(G)** Diagram of NMD magnitude during development *in vitro* and *in vivo*. NMD magnitude increases (NMD target expression decreases with a mirrored increase in NMD factors) during ESC priming *in vitro* and during implantation and EPI expansion *in vivo*.

Reviewer 3:

In this set of well-designed experiments, the authors demonstrate that nonsense-mediated RNA decay (NMD) has stage- and lineage-specific developmental functions in the early mammalian embryo. Previous studies established that loss or depletion of NMD causes early embryo lethality in diverse organisms. However, in mice, the timing and underlying molecular mechanisms of lethality remain unknown. Like in my other biological processes, proteome regulation in early embryonic brain development has been mostly studied at the level of transcription, so mRNA stability has not been demonstrated. In this manuscript, to determine the role of NMD pathway in early mouse embryo development, the authors targeted UPF2, one of the major factors of NMD machinery. They found that UPF2 is dispensable at pre-implantation stage to form the blastocyst but essential peri-implantation. They then performed an RNAseq at this stage and found that genes downregulated upon knockout of Upf2 regulates embryo development and pluripotency. Genes upregulated in *Upf2*-null blastocysts are enriched for apoptosis and cell cycle arrest. Most importantly, the authors identified high-confidence NMD target mRNAs in inner cell mass and epiblast in the blastocyst stage.

© 2022. Published by The Company of Biologists under the terms of the Creative Commons Attribution License (https://creativecommons.org/licenses/by/4.0/).

I only have minor comments:

The last sentence of introduction (…to revealed…) may need correction
Response: This was noted by R1 as well and has been corrected.

Describe the PCA abbreviation when first mentioned
Response: This has been added.

In the discussion, can authors also speculate if disruption of other essential NMD factors would cause similar phenotypes and gene expression changes.
Response: As indicated in response to **Rev 1**, above, we found that disruption of other NMD factors causes embryonic lethality at a similar, if not identical, stage as disruption of UPF2. Unfortunately, the exact timing of lethality was not determined in these earlier studies, nor were specific developmental defects identified. These points have been added to the Discussion. With regard to the question about gene expression, to our knowledge, no study besides ours has performed genome-wide analysis of gene expression in mouse embryos in response to NMD factor knockout. We can add this point to the manuscript if deemed important.

Be consistent with the italic use of *Upf2*-null throughout the text (i.e., Figure 1 legend, first sentence)
Response: We thank the reviewer for catching this instance in which we neglected to italicize *Upf2*. It has been corrected.

We thank all the Reviewers for their thoughtful comments and suggestions to improve our manuscript. We believe our study on the role of RNA turnover in embryo development provides novel insights and presents a unique perspective to the field of developmental biology. Our study will also be of interest to reproductive biologists, RNA biologists, and those in the regenerative medicine/stem cell field.

Second decision letter

MS ID#: DEVELOP/2022/200764

MS TITLE: Progression of the Pluripotent Epiblast Depends Upon the NMD Factor UPF2

AUTHORS: Jennifer N Chousal, Abhishek Sohni, Kristoffer Vitting-Seerup, Kyucheol Cho, Matthew Kim, Kun Tan, Bo Porse, Miles F Wilkinson, and Heidi Cook-Andersen

I have now received all the referees reports on the above manuscript. I am happy to report that the three Reviewers are positive for publication of your manuscript. Thank you for your efforts towards addressing their comments during the revision. The referees' comments are appended below, or you can access them online: please go to BenchPress and click on the 'Manuscripts with Decisions' queue in the Author Area.

There is only one minor point, which I believe would be good to address, when uploading your final text version for publication. Reviewer 2 suggests that you include a few sentences where you disclose the technical limitations, for example, of why you were unable to address any maternal contribution of NMD and why they needed to use ESC derivation from their mutant blastocysts to indirectly address the question of cell survival.

This is just a very minor text addition, but I believe will help the readers to better understand your experimental design and the extent of the conclusions.

I will of course not send your manuscript back to the Reviewers, but it would be great if you could make such changes in the text and submit the final version of your text file with those additions at your earliest convenience, so that we can proceed with publication.

© 2022. Published by The Company of Biologists under the terms of the Creative Commons Attribution License (https://creativecommons.org/licenses/by/4.0/).

Reviewer 1

Advance summary and potential significance to field

I think that is an important paper, not only for the NMD field but also for those interested in early development.

Comments for the author

The authors performed a thorough revision and have addressed the minor concerns and suggestions that I raised during the first round of review. They also seem to have addressed concerns raised by the other two reviewers, Thus, I remain enthusiastic about this study. I do recommend publication in Development.

Reviewer 2

Advance summary and potential significance to field

The authors have done their best to address the reviewers' comments in their revised manuscript and point-by-point responses. As such, and taking into account several technical limitations (e.g. no good anti-UPF2 antibody, or not being able to definitively show that epiblast cells fail to survive in situ in embryos) of the study, the revised submission should proceed to publication. It would however be appropriate were the authors to disclose these limitations, regards e.g. how they were unable to address any maternal contribution and why they needed to use ESC derivation from their mutant blastocysts to indirectly address the question of cell survival.

Comments for the author

The authors have done their best to address the reviewers' comments in their revised manuscript and point-by-point responses. As such, and taking into account several technical limitations (e.g. no good anti-UPF2 antibody, or not being able to definitively show that epiblast cells fail to survive in situ in embryos) of the study, the revised submission should proceed to publication. It would however be appropriate were the authors to disclose these limitations, regards e.g. how they were unable to address any maternal contribution and why they needed to use ESC derivation from their mutant blastocysts to indirectly address the question of cell survival.

Reviewer 3

Advance summary and potential significance to field

I am satisfied with the revision. I had only minor concerns which were addressed by the authors. The authors also followed my suggestions.

Comments for the author

I am satisfied with the revision. I had only minor concerns which were addressed by the authors. The authors also followed my suggestions.

Second revision

Author response to reviewers' comments

Reviewer 1:
I think that is an important paper, not only for the NMD field but also for those interested in early development. The authors performed a thorough revision and have addressed the minor concerns and suggestions that I raised during the first round of review. They also seem to have addressed

© 2022. Published by The Company of Biologists under the terms of the Creative Commons Attribution License (https://creativecommons.org/licenses/by/4.0/).

concerns raised by the other two reviewers, Thus, I remain enthusiastic about this study. I do recommend publication in Development.

We thank the reviewer for their time in reviewing our manuscript and their enthusiasm for our study.

Reviewer 2:
The authors have done their best to address the reviewers' comments in their revised manuscript and point-by-point responses. As such, and taking into account several technical limitations (e.g. no good anti-UPF2 antibody, or not being able to definitively show that epiblast cells fail to survive in situ in embryos) of the study, the revised submission should proceed to publication. It would however be appropriate were the authors to disclose these limitations, regards e.g. how they were unable to address any maternal contribution and why they needed to use ESC derivation from their mutant blastocysts to indirectly address the question of cell survival.

We agree that these limitations should be disclosed in the final publication, and thank the reviewer for this suggestion. As such, we have added the following discussion points:
- Page 12, Line 304-309: “To further assess this EPI defect, we first attempted a number of approaches to directly assay the nature of the EPI progression defect, including TUNEL analysis and immunofluorescence analysis of active caspase 3 expression in NANOG-positive cells during in vitro outgrowth. However, the very low number of NANOG-positive cells in Upf2-null blastocysts and their rapid depletion upon initiation of outgrowth prohibited making accurate quantitative comparisons.”
- Page 18, Lines 453-458: “Of note, it is possible that the phenotype observed in this study was affected by maternal UPF2 inherited from the oocyte. Unfortunately, we could not test this possibility, as the UPF2 antibody available (Lykke-Andersen et al., 2000) is unable to differentiate between endogenous UPF2 and the truncated UPF2 produced by the Upf2-null mutant. However, if true, maternal UPF2 would be expected to mask and/or reduce defects in the embryo, suggesting that the phenotype of Upf2-null mice in this study might be an underestimate of UPF2’s early embryonic functions.”

Reviewer 3:
I am satisfied with the revision. I had only minor concerns which were addressed by the authors. The authors also followed my suggestions.

We thank the reviewer for their time in reviewing our manuscript and their enthusiasm for our study.

Third decision letter

MS ID#: DEVELOP/2022/200764

MS TITLE: Progression of the Pluripotent Epiblast Depends Upon the NMD Factor UPF2

AUTHORS: Jennifer N Chousal, Abhishek Sohni, Kristoffer Vitting-Seerup, Kyucheol Cho, Matthew Kim, Kun Tan, Bo Porse, Miles F Wilkinson, and Heidi Cook-Andersen
ARTICLE TYPE: Research Article

Thank you for attending to the last, minor modifications suggested by Reviewer 2. I am happy to tell you that your manuscript has been accepted for publication in Development, pending our standard ethics checks.

© 2022. Published by The Company of Biologists under the terms of the Creative Commons Attribution License (https://creativecommons.org/licenses/by/4.0/).
